# Silica nanomaterials induce organ injuries by Ca^2+^-ROS-initiated disruption of the endothelial barrier and triggering intravascular coagulation

**DOI:** 10.1186/s12989-020-00340-8

**Published:** 2020-03-23

**Authors:** De-Ping Wang, Zhao-Jun Wang, Rong Zhao, Cai-Xia Lin, Qian-Yu Sun, Cai-Ping Yan, Xin Zhou, Ji-Min Cao

**Affiliations:** 1 Key Laboratory of Cellular Physiology at Shanxi Medical University, Ministry of Education, and the Department of Physiology, Shanxi Medical University, Taiyuan, China; 2 Center of Translational Medicine, Shanxi Medical University, Taiyuan, China; 3 Department of Medical Imaging, Shanxi Medical University, Taiyuan, China

**Keywords:** Silica nanoparticle, Endothelium, Coagulation, Calcium, Reactive oxygen species

## Abstract

**Background:**

The growing use of silica nanoparticles (SiNPs) in many fields raises human toxicity concerns. We studied the toxicity of SiNP-20 (particle diameter 20 nm) and SiNP-100 (100 nm) and the underlying mechanisms with a focus on the endothelium both in vitro and in vivo.

**Methods:**

The study was conducted in cultured human umbilical vein endothelial cells (HUVECs) and adult female Balb/c mice using several techniques.

**Results:**

In vitro, both SiNP-20 and SiNP-100 decreased the viability and damaged the plasma membrane of cultured HUVECs. The nanoparticles also inhibited HUVECs migration and tube formation in a concentration-dependent manner. Both SiNPs induced significant calcium mobilization and generation of reactive oxygen species (ROS), increased the phosphorylation of vascular endothelial (VE)-cadherin at the site of tyrosine 731 residue (pY731-VEC), decreased the expression of VE-cadherin expression, disrupted the junctional VE-cadherin continuity and induced F-actin re-assembly in HUVECs. The injuries were reversed by blocking Ca^2+^ release activated Ca^2+^ (CRAC) channels with YM58483 or by eliminating ROS with N-acetyl cysteine (NAC). In vivo, both SiNP-20 and SiNP-100 (i.v.) induced multiple organ injuries of Balb/c mice in a dose (range 7–35 mg/kg), particle size, and exposure time (4–72 h)-dependent manner. Heart injuries included coronary endothelial damage, erythrocyte adhesion to coronary intima and coronary coagulation. Abdominal aorta injury exhibited intimal neoplasm formation. Lung injuries were smaller pulmonary vein coagulation, bronchiolar epithelial edema and lumen oozing and narrowing. Liver injuries included multifocal necrosis and smaller hepatic vein congestion and coagulation. Kidney injuries involved glomerular congestion and swelling. Macrophage infiltration occurred in all of the observed organ tissues after SiNPs exposure. SiNPs also decreased VE-cadherin expression and altered VE-cadherin spatial distribution in multiple organ tissues in vivo. The largest SiNP (SiNP-100) and longest exposure time exerted the greatest toxicity both in vitro and in vivo.

**Conclusions:**

SiNPs, administrated in vivo, induced multiple organ injuries, including endothelial damage, intravascular coagulation, and secondary inflammation. The injuries are likely caused by upstream Ca^2+^-ROS signaling and downstream VE-cadherin phosphorylation and destruction and F-actin remodeling. These changes led to endothelial barrier disruption and triggering of the contact coagulation pathway.

## Background

Silica (silicon dioxide, SiO_2_) is one of the most plentiful compounds in the earth crust [1] and is a useful material in many industrial fields. Some silica present in nano-scale (10^− 9^ m) spherical shape and are called silica nanoparticles (SiNPs or SiO_2_-NPs). SiNPs may naturally exist or be added to products by humans during fabrication or manufacturing. SiNPs have wide applications because of their unique properties and have been used in fields such as microelectronics [2], material science and industry [3], agriculture, food and consumer products (including cosmetics) [4–7], and medicine (drug delivery, diagnostic and medicine imaging and engineering) [8, 9]. However, large-scale industrial SiNPs production and global SiNPs commercialization have increased human exposure risks [10].

SiNPs are generally considered harmful to human health, but it is unclear exactly how SiNPs affect cells, tissues, and organs. Murugadoss et al. [10] reviewed and summarized the cytotoxicity and mode of action of SiNPs caused by different routes of exposure. The cellular mechanisms include DNA damage, cell cycle arrest, immunotoxicity, autophagy, oxidative stress, and dysfunction of endothelial cells and blood cells. Cell type is another variable related to SiNPs cytotoxicity. They also summarized the in vivo toxicity of SiNPs and factors that affect the toxicity [10], such as the exposure time (single or multiple) and duration (acute or chronic), route of exposure (ingestion, inhalation, dermal contact, parenteral injection), physico-chemical characterization of SiNPs (primary size, shape, crystallinity, and chemical composition or purity), synthetic process (thermal or wet route), and types of SiNPs (colloidal, amorphous, hydrophilic or hydrophobic). Alternative mechanisms may cause SiNPs cytotoxicity including endoplasmic reticulum stress-associated apoptosis [11], mitochondrial pathway-mediated apoptosis [12], reactive oxygen species (ROS)-associated intracellular acidosis [13], and transient receptor potential (TRP) ion channel-associated event [14].

Leong’s group [15–18] demonstrated the toxicity of inorganic nanoparticles to vascular endothelial (VE)-cadherin, a critical adherens junction protein which participates in the integrity of endothelial barrier/permeability, in the cytotoxicity of inorganic nanoparticles. Titanium dioxide nanomaterials induced endothelial leakiness [15] and accelerated both intravasation and extravasation of breast cancer cells by disrupting the homophilic interaction of VE-cadherin [16]. Gold nanoparticles induced endothelial leakiness by VE-cadherin phosphorylation and expression downregulation [17]. Mesoporous SiNPs directly increased endothelial permeability by perturbing VE-cadherin-based endothelial cell-cell adhesion in a gravitational way [18]. The researchers created mesoporous SiNPs with different densities by backfilling the mesopore with pre-determined amount of tetraethoxysilane (TEOS) while maintaining a constant particle size. Heavier SiNPs (with greater density and thus larger gravitational force) induced greater damage to the VE-cadherin adherens junctions than lighter SiNPs [18]. These results indicate the importance of endothelial junctional adhesions in the toxicity of nanoparticles. There are few additional studies of the toxicity, and modes of action. Using human umbilical vein endothelial cells (HUVECs) in vitro and mice in vivo, we evaluated the possibility that SiNPs may induce multiple organ injuries by perturbing VE-cadherin adherens junctions via the Ca^2+^-ROS signaling pathway. Results from several approaches proved the above possibility. The study may help to better understand the biotoxicity of SiNPs and to develop associated translational approaches.

## Results

### Characterization of SiNPs

**Fig. 1:**
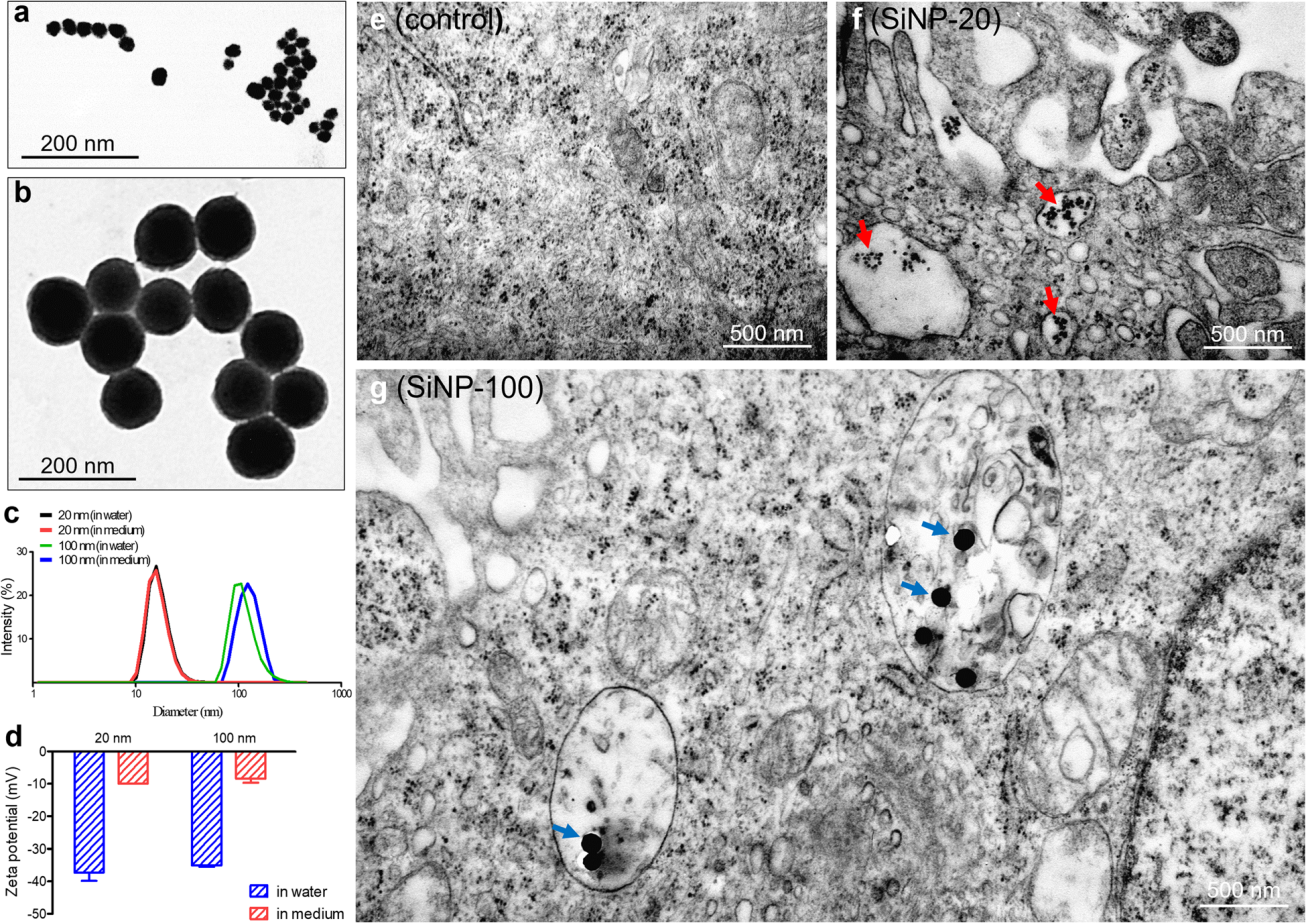
Characterization and endocytosis of SiNPs. **a** and **b**, TEM images of SiNP-20 and SiNP-100. **c**, hydrodynamic diameters of SiNP-20 and SiNP-100 respectively in water and culture medium. **d**, zeta potentials of SiNP-20 and SiNP-100 respectively in water and culture medium. **e**, **f,** and **g**, TEM images showing the internalization of SiNP-20 and SiNP-100 in cultured HUVECs after exposure for 24 h. Control (**e**), before exposure to SiNPs. Note that the endocytosed SiNP-20 (red arrows) (**f**) and SiNP-100 (blue arrows) (**g**) were presented inside cell vesicles

The morphological features of SiNPs in the culture medium were examined using transmission electron microscopy (TEM). Two sizes of SiNPs were used in the study: 20 nm diameter (SiNP-20) and 100 nm diameter (SiNP-100). TEM images showed that SiNPs were spherical and had uniform sizes (Fig. 1a, b). Dynamic light scattering (DLS) experiments showed that the hydrodynamic sizes of SiNP-20 and SiNP-100 were respectively 35.4 ± 1.2 nm and 120.8 ± 1.0 nm in water, and were 40.2 ± 1.5 nm and 160.2 ± 1.4 nm in serum-containing culture medium (Fig. 1c). Zeta potential testing showed that the surfaces of the SiNPs, in water, were negatively charged as –36.7 ± 2.7 mV and –35.1 ± 0.5 mV, respectively, for SiNP-20 and SiNP-100. They became –9.8 ± 0.2 mV and –8.5 ± 1.3 mV in serum-containing culture medium (Fig. 1d), indicating a differential surface charge and overall good particle stability.

### Cell study

#### Uptake of SiNPs by endothelial cells

The uptake of SiNPs by cultured human umbilical vein endothelial cells (HUVECs) and their intracellular distribution were visualized with TEM. Control HUVECs (without SiNPs exposure) showed a normal structure (Fig. 1e). Endocytosis of SiNP-20 and SiNP-100 (both at 50 μg/mL) by HUVECs was observed 24 h after exposure to the nanoparticles. The internalized SiNPs of both sizes were mainly localized inside the endosome-like vesicles (Fig. 1f, g) and matched the scale. SiNPs were not found in other organelles.

#### SiNPs decreased the viability and damaged the plasma membrane of HUVECs

**Fig. 2:**
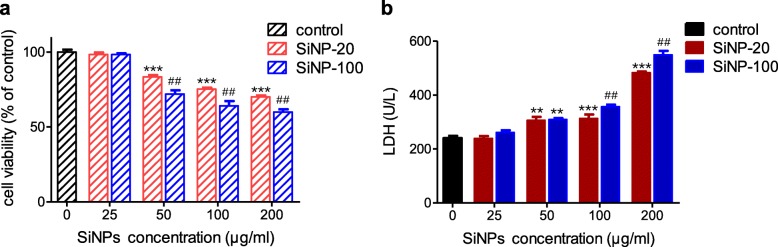
The toxic effects of SiNPs on cell viability and membrane integrity in cultured HUVECs. **a**, CCK8 assay showing the effects of SiNPs on cell viability (*n* = 3 independent experiments). Both SiNP-20 and SiNP-100 decreased the viability of HUVECs in a concentration (0–200 μg/mL)-dependent manner, and SiNP-100 was more toxic than SiNP-20. *** *p* < 0.001 vs. control. ^##^*p* < 0.01 vs. SiNP-20 at the same concentration. **b**, LDH assay showing the toxic effects of SiNPs on the membrane integrity as indicated by LDH release from HUVECs. Both SiNP-20 and SiNP-100 increased LDH release in a concentration (0–200 μg/mL)-dependent manner, and SiNP-100 was more toxic than SiNP-20 on the membrane. ** *p* < 0.01 vs. control, *** *p* < 0.001 vs. control. ^##^*p* < 0.01 vs. SiNP-20 at the same concentration

The cytotoxicity of SiNPs was evaluated using a CCK8 assay 24 h after exposure of cultured HUVECs to SiNP-20 and SiNP-100 at concentrations of 0 (control), 25, 50, 100 and 200 μg/mL. Both sizes of SiNPs reduced the cell viability in a concentration-dependent manner (*p* < 0.001 vs. control) (Fig. 2a). The minimal toxic concentration of both SiNP-20 and SiNP-100 was 50 μg/mL. At 50–200 μg/mL concentrations, SiNP-100 was more toxic to cell viability than SiNP-20 (*p* < 0.01) (Fig. 2a). Exposure to SiNP-20 and SiNP-100 for 24 h increased LDH release from HUVECs in a concentration-dependent manner (*p* < 0.01 vs. control) (Fig. 2b). SiNP-100, at 100 μg/mL and 200 μg/mL, was more toxic in inducing LDH release than SiNP-20 at the same concentrations (*p* < 0.01) (Fig. 2b). These results show that both SiNPs decreased the viability of HUVECs and damaged the membrane integrity. The cytotoxicity of SiNP-100 was greater than SiNP-20.

#### SiNPs induced calcium mobilization in HUVECs

We used two techniques to investigate the potential effects of SiNPs on the calcium homeostasis of HUVECs. The MetaFluor intracellular Ca^2+^ imaging system (Universal Imaging, Cairn Research, Kent, UK) detected calcium transient/mobilization and a non-invasive micro-test (NMT) (Younger, Amherst, USA) monitored Ca^2+^ flux across the plasma membrane in a real-time fashion. These two methods demonstrated the effects of SiNPs on the calcium dynamics of endothelial cells.

**Fig. 3:**
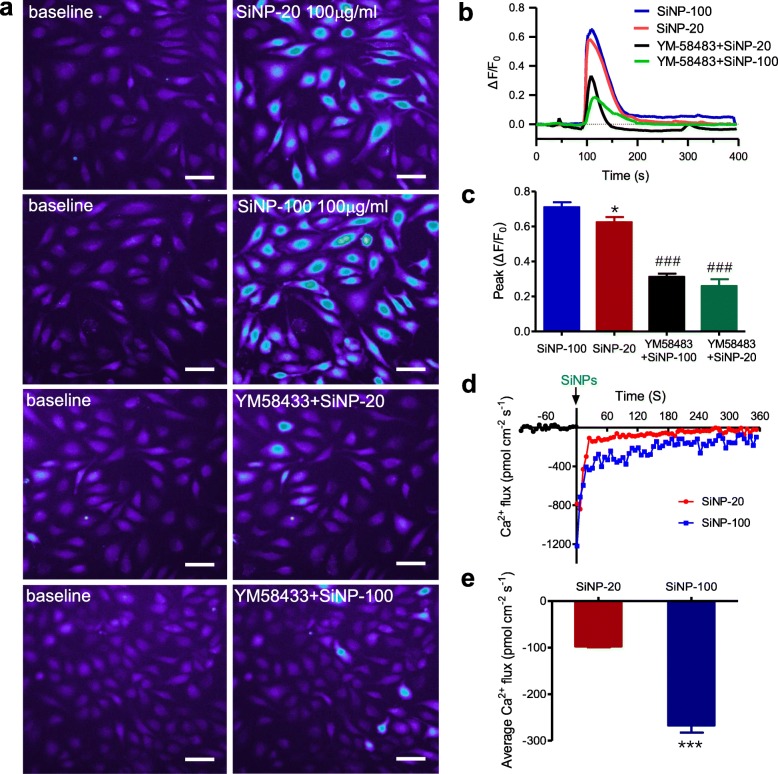
The calcium mobilizing effects of SiNPs in HUVECs. **a**, representative intracellular free Ca^2+^ images. Compared with the baseline, both SiNP-20 and SiNP-100 at 100 μg/mL exposing for about 120 s induced significant [Ca^2+^]_i_ elevation which suggests calcium mobilization. Blocking CRAC channel with YM58433 abolished these effects of SiNP-20 and SiNP-100. **b**, time course of ΔF/F0 ratio. **c**, statistical [Ca^2+^]_i_ peak (ΔF/F0) (*n* = 3 independent experiments). Note that both SiNP-20 and SiNP-100 induced significant calcium transients, and SiNP-100 was more potent than SiNP-20 in this effect. * *p* < 0.05, SiNP-20 vs. SiNP-100. ^###^*p* < 0.001, SiNPs+YM58483 vs. SiNPs alone. **d**, results of non-invasive micro-test (NMT) showing abrupt induction of Ca^2+^ influx by either SiNP-20 or SiNP-100 (100 μg/mL) in HUVECs. Downward currents represented Ca^2+^ influx while upward currents represented Ca^2+^ efflux in this method. **e**, statistical results of NMT indicating the average Ca^2+^ influx induced by SiNP-20 and SiNP-100. SiNP-100 induced more Ca^2+^ influx than SiNP-20 (*** *p* < 0.001), *n* = 4 cells for each treatment

Intracellular Ca^2+^ imaging results showed that acute exposure of HUVECs to SiNP-20 or SiNP-100 at 100 μg/mL induced a rapid rise of intracellular free Ca^2+^ level in HUVECs as indicated by rapid increases of Ca^2+^ fluorescence intensity ΔF/F0 (Fig. 3a, b) and peak ΔF/F0 (Fig. 3c). After obtaining a baseline recording at 100 s, a faster and larger calcium transient was recorded with a time-to-peak within 60 s, cells pre-loaded with Fura-4/AM lighted up within 90 s after exposure to SiNP-20 or SiNP-100 (Fig. 3a-c). Both the magnitude (ΔF/F0) (Fig. 3b) and the peak value of Ca^2+^ transients (peak ΔF/F0) (Fig. 3c) were greater for SiNP-100 than that those for SiNP-20. Pretreatment with YM58483 (also named BTP2) (10 μmol/L), a potent blocker of Ca^2+^ release activated Ca^2+^ (CRAC) channels, significantly reduced the amplitude of Ca^2+^ transients induced by both SiNPs (Fig. 3a-c). These results indicate that SiNPs at 100 μg/mL induce Ca^2+^ mobilization and this is done, at least partially, by activating CRAC channels.

NMT experiments were conducted by placing a platinum-tipped microelectrode near the surface of HUVECs to measure the Ca^2+^ generated in the cell’s vicinity along axes perpendicular to the cell surface. A downward current trace below the X axis indicated Ca^2+^ influx and an upward trace indicated Ca^2+^ efflux. Using this approach, we first obtained a baseline recording of Ca^2+^ flux in the HUVEC membrane for about 60 s, and then directly observed that both SiNPs triggered a rapid Ca^2+^ influx (Fig. 3d). Measurement of total Ca^2+^ flux over 360 s after SiNPs exposure revealed that SiNPs induced significant Ca^2+^ influx in HUVECs compared to the baseline (Fig. 3d). Ca^2+^ influx returned nearly to baseline within 360 s, after SiNP-20 or SiNP-100 (both at 100 μg/mL) exposure (Fig. 3d). SiNP-100 induced greater Ca^2+^ influx than SiNP-20 (*p* < 0.001) (Fig. 3e). Blank control (without cell) and positive control (high K^+^, 60 mmol/L) results of the NMT tests are shown in Figure S1.

#### SiNPs induced intracellular ROS generation in HUVECs

**Fig. 4:**
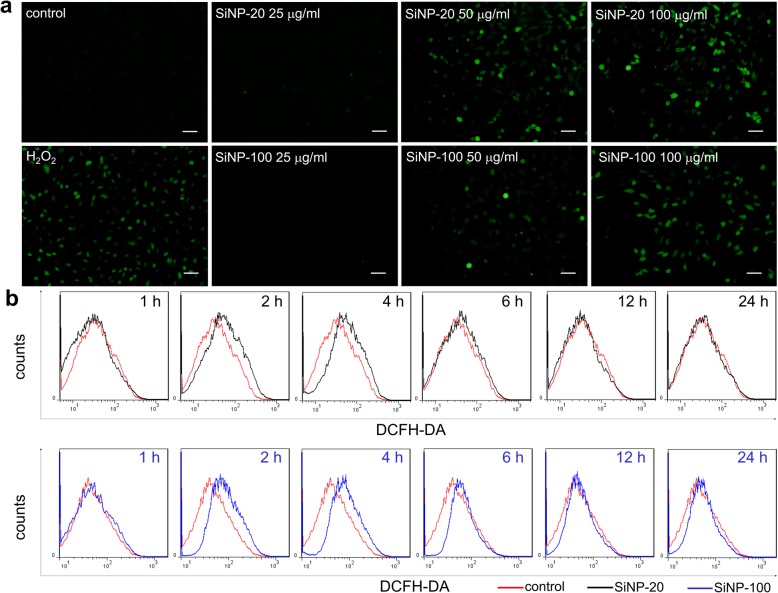
Induction of intracellular ROS generation by SiNPs in HUVECs. **a**, representative ROS images (green) measured with fluorescence microscopy. Both SiNP-20 and SiNP-100 induced ROS generation in a concentration (0–100 μg/mL)-dependent manner. As a positive control, H_2_O_2_ (7.5 mg/mL) increased the ROS level in HUVECs. Scale bar = 50 μm. **b**, flow cytometry assay for ROS detection, which showed the time course of ROS generation in HUVECs exposed to SiNPs at a fixed concentration (100 μg/mL). A rightward shift of the trace indicated ROS elevation. Both SiNP-20 and SiNP-100 induced significant ROS generation especially at 2–4 h after exposure. Counts, cell numbers

Calcium mobilization can trigger intracellular ROS generation [19], and ROS may underlie the toxic effects of nanoparticles [20]. We therefore examined whether SiNPs would induce ROS generation in HUVECs. Results of fluorescence microscopy showed that both SiNP-20 and SiNP-100 significantly increased intracellular ROS level in HUVECs in a concentration dependent manner (Fig. 4a) with a minimal effective concentration of 50 μg/mL (Fig. 4a). As a positive control, H_2_O_2_ (7.5 mg/mL) strongly increased the ROS level (Fig. 4a).

We used flow cytometry to study the effects of SiNP-20 and SiNP-100 on ROS generation in HUVECs over time at a fixed concentration (100 μg/mL). Both SiNP-20 and SiNP-100, especially at 2–4 h after exposure, significantly increased the intracellular ROS levels compared to the control (Fig. 4b). SiNP-100 exerted a greater stimulating effect on ROS generation than SiNP-20 in this approach (Fig. 4b). After 2 h of exposure the ROS levels were increased by 1.55 and 1.92 times respectively by SiNP-20 and SiNP-100 compared to their control values.

#### SiNPs inhibited HUVECs migration and tube formation

**Fig. 5:**
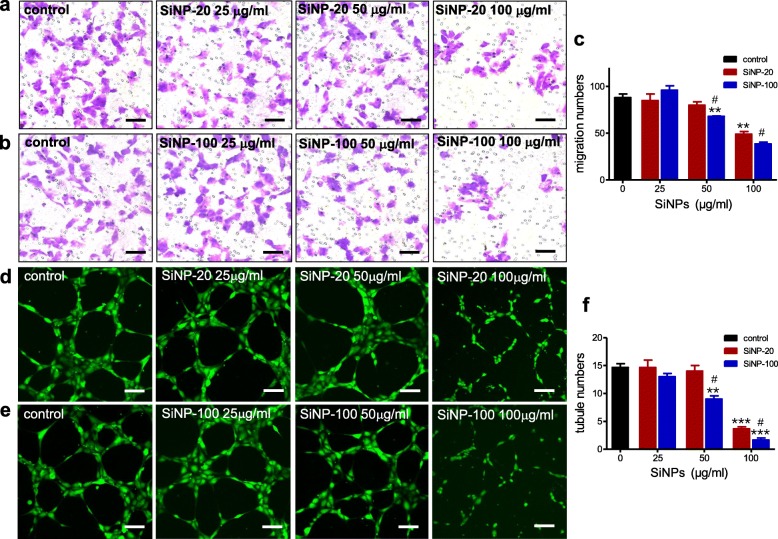
The inhibitory effects of SiNPs on endothelial migration and tube formation. a, b and c, transwell assays showing the effects of SiNP-20 (**a**) and SiNP-100 (**b**) on the migration of HUVECs. **c**, statistical results of migration (*n* = 3 independent assays). Both SiNP-20 and SiNP-100 decreased cell migration roughly in a concentration (0–100 μg/mL)-dependent manner, and SiNP-100 was more potent than SiNP-20 in suppressing the migration. ** *p* < 0.01 vs. control. ^#^*p* < 0.05 vs. SiNP-20. **d**, **e** and **f**, confocal results showing the inhibitory effects of SiNP-20 (**d**) and SiNP-100 (**e**) on the tube formation of HUVECs. Statistical results of tube numbers were shown in (**f**) (*n* = 3 independent assays). Both SiNP-20 and SiNP-100 suppressed the tubule formation in a concentration (0–100 μg/mL) dependent manner. SiNP-100 was more toxic than SiNP-20 on the tube formation. ** *p* < 0.01, *** *p* < 0.001 vs. control. ^#^*p* < 0.05 vs. SiNP-20. Scale bar = 50 μm

Endothelial cell migration is a key process in the formation of endothelial barrier. Transwell assays were used to determine whether SiNPs could affect endothelial migration. The concentrations of SiNP-20 and SiNP-100 tested were 0, 25, 50 and 100 μg/mL. Both SiNP-20 (Fig. 5a) and SiNP-100 (Fig. 5b) at 100 μg/mL almost ended HUVECs migration roughly after exposure for 12 h. SiNP-100 was more potent than SiNP-20 in inhibiting endothelial cell migration (*p* < 0.01 vs. SiNP-20 at 50 μg/mL) (Fig. 5c).

Endothelial cells can form tubule-like structures in appropriate media, and tubule formation is another key step in angiogenesis and the formation of the endothelial barrier. We used a three-dimensional matrigel assay (Corning Life Sciences, Acton, MA, USA) to examine the potential effects of SiNPs on the tube formation of HUVECs. When HUVEC cells were seeded into the matrigel, robust and elongated tube-like structures gradually formed under the control condition (Fig. 5d, e). Exposure to SiNP-20 (Fig. 5d) and SiNP-100 (Fig. 5e) for 6 h significantly inhibited the tube formation of HUVECs roughly in a concentration (0–100 μg/mL) dependent manner. The numbers of formed tubules were calculated and shown in Fig. 5f. SiNP-100 was more potent than SiNP-20 in inhibiting the tube formation (*p* < 0.05 at 50 and 100 μg/mL).

#### SiNPs decreased the expression of VE-cadherin, disrupted the junctional integration of VE-cadherin and induced F-actin re-assembly in HUVEC cultures

**Fig. 6:**
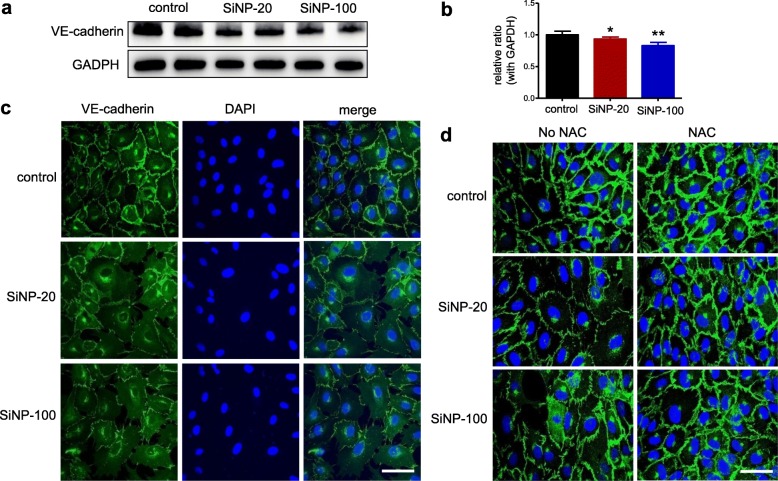
The inhibitory effects of SiNPs on the expression and junctional continuity of VE-cadherin in HUVECs. **a** and **b**, western blots of VE-cadherin in HUVECs before (control) and after exposure to SiNPs (100 μg/mL) for 24 h and the semi-quantitative results, respectively (*n* = 3). Both SiNP-20 and SiNP-100 decreased VE-cadherin expression. * *p* < 0.05, ** *p* < 0.01 vs. control. **c**, confocal images of VE-cadherin in HUVECs culture. VE-cadherin presented at the cell-cell junctions. Both SiNP-20 and SiNP-100 exposure for 2 h deteriorated the conformation and integration of VE-cadherin in the endothelial junctions, and VE-cadherin even disappeared at some junctional areas. Scale bar = 20 μm. **d**, confocal images showing that NAC (a ROS scavenger) improved the SiNPs (2 h)-induced changes of VE-cadherin in HUVECs cultures. In addition, HUVECs exposed to SiNPs for 2 h showed larger sizes compared with control HUVECs, and NAC restored the cell size. Scale bar = 20 μm

Western blotting results showed that exposure of HUVECs to both SiNPs at 100 μg/mL significantly decreased the expression of VE-cadherin in HUVEC cultures compared to control cells (*p* < 0.05 for SiNP-20 and *p* < 0.01 for SiNP-100) (Fig. 6a, b). Application of SiNP-20 and SiNP-100 reduced VE-cadherin protein expression by 7 and 17%, respectively (Fig. 6b). Confocal results show that control HUVECs (not exposed to SiNPs) had clear, tight and continuous VE-cadherin distributions in cell-cell junctions. After exposure to SiNP-20 or SiNP-100 (100 μg/mL) for 2 h, VE-cadherin protein expressions declined and lost their continuity (Fig. 6c). The presence of NAC (a ROS scavenger) in the HUVECs culture attenuated SiNPs-induced disruption of junctional VE-cadherin continuity (Fig. 6d). This result suggested the role of ROS in SiNPs-induced endothelial barrier destruction. In addition, HUVECs had larger cell size after exposure to SiNPs, and NAC largely restored (reduced) the cell size (Fig. 6d).

**Fig. 7:**
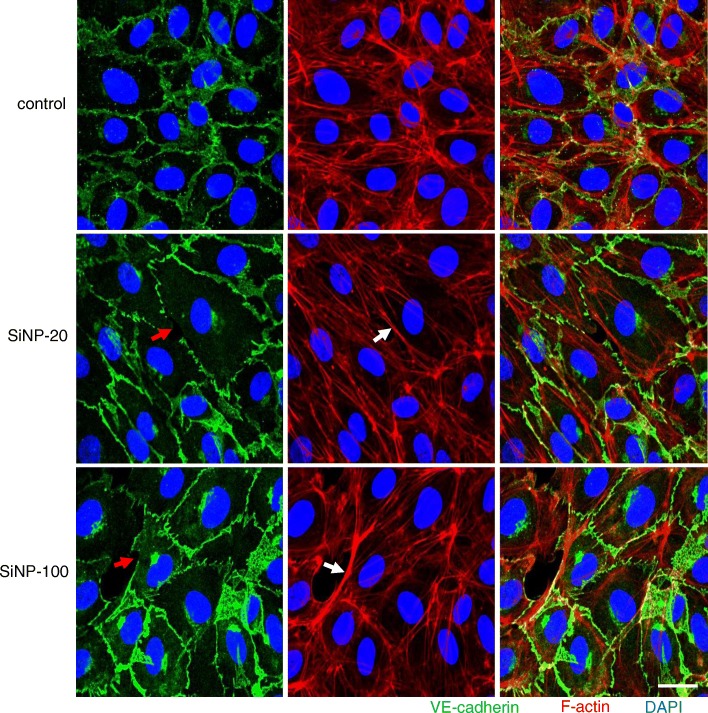
Confocal images exhibiting the effects of SiNPs on the junctional integrity of VE-cadherin (green) and assembly of F-actin (red) in HUVEC cultures. Nuclei were stained blue by DAPI. HUVECs monolayer showed normal distribution of VE-cadherin and F-actin before exposure to SiNPs (upper row). After exposure to SiNP-20 (100 μg/mL), HUVECs monolayer showed larger cell size, discontinued junctional pattern of VE-cadherin (red arrow) and re-assembly of F-actin (white arrow) which suggests F-actin remodeling (middle row). SiNP-100 exerted similar effects on cell size, VE-cadherin continuity (red arrow) and F-actin re-assembly (white arrow), and was likely stronger than SiNP-20 in inducing F-actin remodeling (lower row). Right column was the merged images of VE-cadherin and F-actin. Scale bar = 10 μm

We also examined the spatial distribution and co-localization of VE-cadherin and F-actin in HUVEC cultures before and after exposure to SiNPs. Compared to the control HUVEC cultures, HUVEC monolayers exposed to SiNP-20 and SiNP-100 had discontinued pattern of VE-cadherin junctional distribution (Fig. 7, left column) and re-assembly of F-actin (Fig. 7, middle column). The merged images show the spatial relation between VE-cadherin and F-actin (Fig. 7, right column). These results suggest involvement of F-actin re-assembly in SiNPs-induced disruption of the endothelial barrier. Again, HUVECs exposed to SiNPs exhibited larger cell size (Fig. 7). SiNP-100 appeared to be more active than SiNP-20 in inducing F-actin re-assembly (Fig. 7).

#### SiNPs increased the phosphorylation of VE-cadherin at the tyrosine 731 (Y731) site in HUVEC cultures

**Fig. 8:**
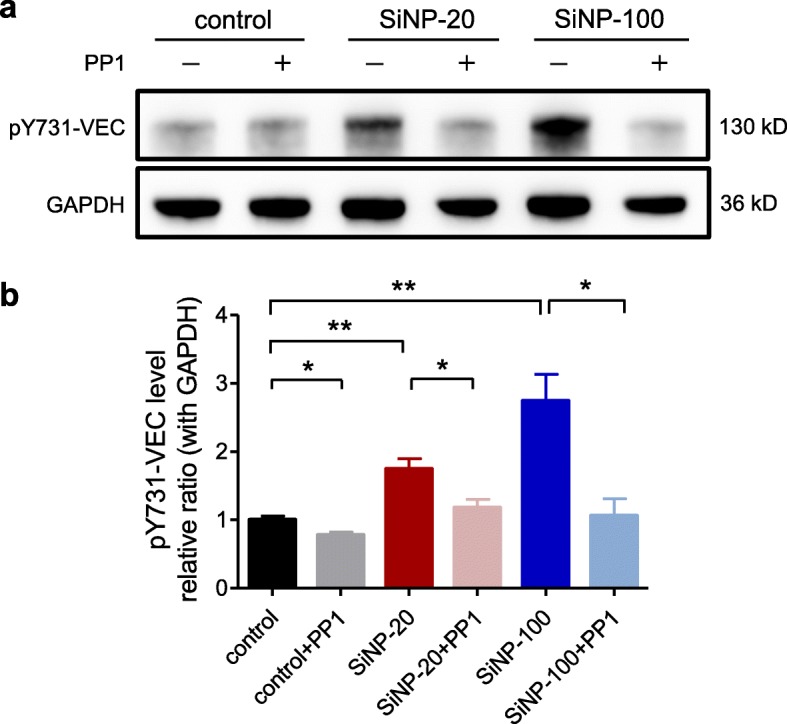
Western blotting showing the levels of phosphorylated VE-cadherin at tyrosine 731 residue (pY731-VEC) in HUVECs at different conditions. **a**, representative electrophoretic bands of western blots. **b**, semiquantitative analysis of pY731-VEC levels. Both SiNP-20 and SiNP-100 (100 μg/mL) exposure for 6 h significantly increased the level of pY731-VEC. * *p* < 0.05, ** *p* < 0.001 vs. control, respectively for SiNP-20 and SiNP-100. This effect was abolished by PP1, a selective inhibitor of Src family tyrosine kinase (* *p* < 0.05 vs. SiNP-20 or SiNP-100 alone). These results suggested that SiNPs increased the phosphorylation of VE-cadherin via activating Src tyrosine kinase

Western blots showed that both SiNP-20 and SiNP-100 exposure for 6 h significantly increased the levels of phosphorylated VE-cadherin at the site of tyrosine 731 residue (pY731-VEC) in HUVECs compared to the control pY731-VEC level (Fig. 8a, b). This SiNPs effect was eliminated by inhibition of Src family tyrosine kinase with PP1 (Fig. 8).

#### Larger SiNPs diffused more onto the cell surface than smaller SiNPs over time based on the ISDD model in vitro

An in vitro sedimentation, diffusion and dosimetry (ISDD) model was used to estimate the SiNPs sedimentations onto the cell surface over time in vitro. The model predicted 43% of the applied dose for SiNP-20 sedimented to the cell surface, and 52% for SiNP-100 over 24 h (Figure S2). The results suggested that larger SiNPs (SiNP-100) reached to the cell surface more than smaller SiNPs (SiNP-20) over time. Based on the ISDD computational estimation, larger SiNPs thus have a higher effective exposure concentration than smaller SiNPs.

### Animal study

#### In vivo exposure to SiNPs induced endothelium-centered multiple organ injuries in mice

The cellular experiments suggested that SiNPs could damage the endothelial barrier via the Ca^2+^-ROS-VE-cadherin signaling pathway. To determine whether SiNPs could cause organ damage through similar mechanisms, we performed in vivo experiments using mice. SiNP-20 and SiNP-100 were intravenously (i.v.) injected into adult female Balb/c mice at bolus doses of 0 (control), 7, 21 and 35 mg/kg (diluted in saline, total 200 μL per injection) based on earlier studies [21–23]. At 4, 24 and 72 h after injection, hearts, aortae, lungs, livers and kidneys were harvested, developed and stained to determine the morphological changes after exposure. The organ injuries induced by SiNPs were related to dose, exposure time, and nanoparticle size. Longer exposure (72 h) to SiNPs induced more significant injuries. We provide the in vivo results obtained after exposure to a dose series of SiNPs (7, 21 and 35 mg/kg, i.v.) for 72 h unless otherwise specified. Organ damages induced by lower doses (7 and 21 mg/kg) of SiNPs exposure for 72 h are shown in Figures S3 and S4 in the Supplementary Material.

##### Heart and aorta injuries

**Fig. 9:**
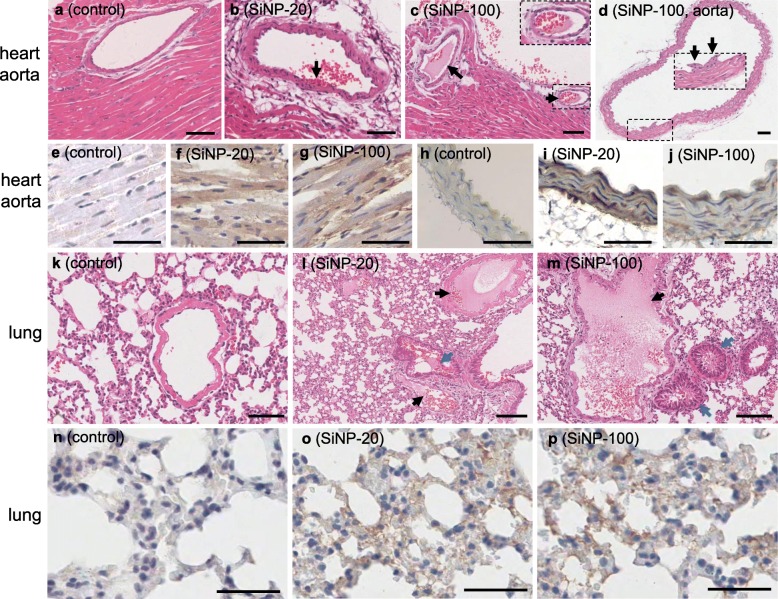
Morphological manifestation of the toxic effects of SiNPs (35 mg/kg, i.v.) on the heart, aorta and lung in mice in vivo. **a**, normal control myocardium (H&E stain). **b**, SiNP-20 induced adherence of red blood cells to the luminal side of a coronary artery (black arrow). **c**, SiNP-100 induced intracoronary coagulation (black arrows), clog shrinkage was seen inside the coronary artery (inset). **d**, SiNP-100 induced cell-containing neoplasm formation in the luminal side of an abdominal aorta (black arrows in the inset which was an enlargement of the framed area). **e**-**j**, immunohistochemical stains of F4/80 (a macrophage marker) in the myocardium (**e**, **f**, **g**) and abdominal aorta (**h**, **i**, **j**) of mice in vivo. Note that F4/80 was negative in control myocardium and aortic wall, but was positive (brown) in mice exposed to SiNP-20 or SiNP-100 for 72 h, suggesting macrophage infiltration and inflammation in these tissues. **k**, **l** and **m**, H&E stains of control lung tissue (**k**) and lung tissues from mice exposed to SiNP-20 (**l**) or SiNP-100 (**m**) for 72 h. Both SiNP-20 and SiNP-100 induced pulmonary vein coagulation (black arrows in panels l and m) and bronchiolic lumen oozing, coagulation and narrowing (blue arrows in panels l and m). **n**, **o** and **p**, immunohistochemical stains of F4/80 in lung tissues, indicating that SiNP-20 and SiNP-100 induced macrophage infiltration in the lung. Scale bar = 100 μm

Hematoxylin and eosin (H&E) stains showed that SiNP-20 at 7 mg/kg did not induce obvious cardiac and aortic injuries, but SiNP-20 at 21 mg/kg induced erythrocyte aggregation and clogging of smaller coronary arteries (Figure S3). SiNP-100 at 7 mg/kg induced erythrocyte aggregation in coronary veins, and at 21 mg/kg induced erythrocyte aggregation and clog in smaller coronary veins (Figure S3). Both SiNPs, at 35 mg/kg, caused serious cardiac injuries mainly characterized by endothelial injury and red blood cell adhesion to the coronary intima (Fig. 9b) and intracoronary coagulation (Fig. 9c) compared to normal control cardiac tissue (Fig. 9a). Coronary clog shrinkage was observed 72 h after injection (Fig. 9c). Surprisingly, cell-containing neoplasm was seen in the intimal side of the abdominal aorta after exposure to the high dose (35 mg/kg) of SiNP-100 (Fig. 9d). Both SiNPs, in a dose-dependent manner, induced macrophage infiltration in the myocardium (Figure S4, first row; Fig. 9f, g) and coronary artery wall (Fig. 9i, j) compared to control myocardium (Fig. 9e) and control aorta (Fig. 9h), as indicated by the positive stains of the macrophage marker F4/80 (brown color).

##### Lung injury

H&E stains showed that SiNP-20 at 7 mg/kg did not significantly damage the lung, but a 21 mg/kg dose induced pulmonary congestion and erythrocyte aggregation in the pulmonary veins (Figure S3). SiNP-100 at 7 mg/kg induced significant erythrocyte aggregation and clog in smaller pulmonary arteries; a 21 mg/kg dose caused serious pulmonary vein congestion and coagulation (Figure S3). Both SiNPs, at 35 mg/kg, induced striking lung injuries characterized by pulmonary congestion and pulmonary vein coagulation, bronchiolar epithelial edema, bronchiolar lumen oozing, narrowing and occlusion (Fig. 9l, m) compared to the control lung tissue (Fig. 9k). SiNP-100 appeared more toxic than SiNP-20 to the lung, because SiNP-100 induced more serious bronchiolar lumen narrowing and pulmonary vein coagulation (Fig. 9m) than SiNP-20 (Fig. 9l). Both SiNPs had a dose-dependent effect in induction of macrophage infiltration (F4/80 positive stains, brown) in the lung (Figure S4; Fig. 9o, p) which suggests pulmonary inflammation.

##### Liver injury

**Fig. 10:**
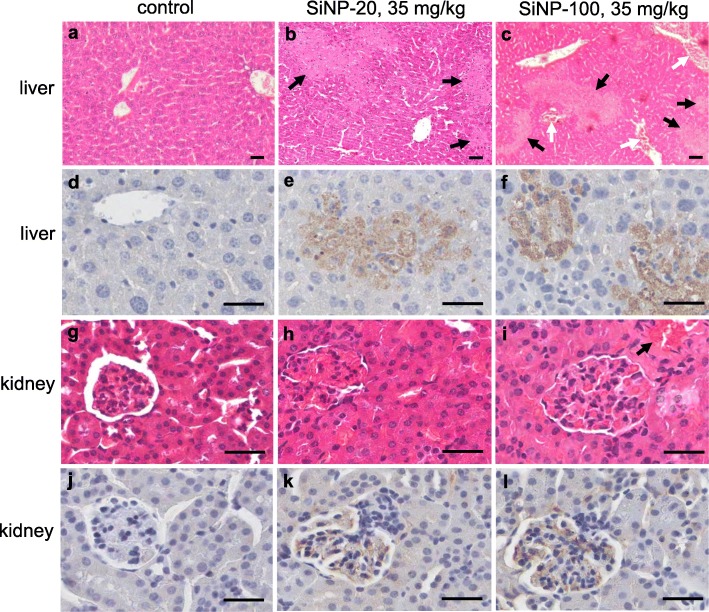
Morphological features reflecting the toxicities of SiNPs (35 mg/kg, i.v.) on the liver and kidney in mice in vivo. a, b and c, H&E stains of liver tissues respectively for control (**a**), SiNP-20 exposure (**b**) and SiNP-100 exposure (**c**) for 72 h. Compared to the control liver tissue, both SiNP-20 and SiNP-100 induced multifocal liver necrosis (black arrows). SiNP-100 induced smaller liver vein congestion and coagulation (white arrows) except for causing focal necrosis. **d**, **e** and **f**, immunohistochemical stains of F4/80 in liver tissues (brown color) which suggested macrophage infiltration after exposure to both SiNPs. **g**, **h** and **i**, H&E stains of kidney tissues. Control kidney tissue showed a normal structure. Both SiNP-20 and SiNP-100 induced glomerular congestion and swelling as reflected by renal capsule narrowing. SiNP-100 also induced hyalinization in some renal areas (red colors indicated by black arrow). **j**, **k** and **l**, immunohistochemical stains of F4/80 in renal tissues which suggested macrophage infiltration after exposure to SiNPs for 72 h. Scale bar = 50 μm

SiNP-20 at 7 mg/kg did no obvious damage to the liver structure (Figure S3), but induced mild erythrocyte congestion and coagulation in the central vein of the liver. SiNP-100 at 7 and 21 mg/kg induced liver vein congestion and coagulation in the central vein of the liver (Figure S3). Both SiNP-20 and SiNP-100, at 35 mg/kg doses for 72 h, induced serious liver injury characterized by liver structural disorder, multifocal necrosis, smaller hepatic vein congestion and coagulation (Fig. 10b, c) compared to the control liver tissue (Fig. 10a). SiNP-100 was relatively more toxic than SiNP-20 to the liver, and induced more serious liver vein coagulation (Fig. 10c) than SiNP-20 (Fig. 10b). Both SiNPs induced macrophage infiltration (F4/80 positive stains, brown) (Figure S4; Fig. 10e, f) in a dose-dependent manner.

##### Kidney injury

SiNP-20 at 7 mg/kg did no obvious damage to the kidney tissue, but at 21 mg/kg, it induced kidney tissue edema and smaller kidney vein congestion (Figure S3). SiNP-100 at 7 mg/kg induced kidney tissue edema and erythrocyte aggregation in smaller kidney veins, and at 21 mg/kg, it caused kidney tissues edema and coagulation in smaller kidney veins (Figure S3). Both SiNPs at 35 mg/kg induced significant kidney injury characterized by glomerular congestion and swelling (Fig. 10h, i) compared with control (Fig. 10g). Both SiNPs induced glomerular macrophage infiltration in a dose-dependent manner (Figure S4; Fig. 10k, l) compared to the control kidney tissue (Figure S4, bottom row; Fig. 10j).

#### SiNPs decreased the expression and altered the spatial distribution of VE-cadherin in multiple organs in vivo

**Fig. 11:**
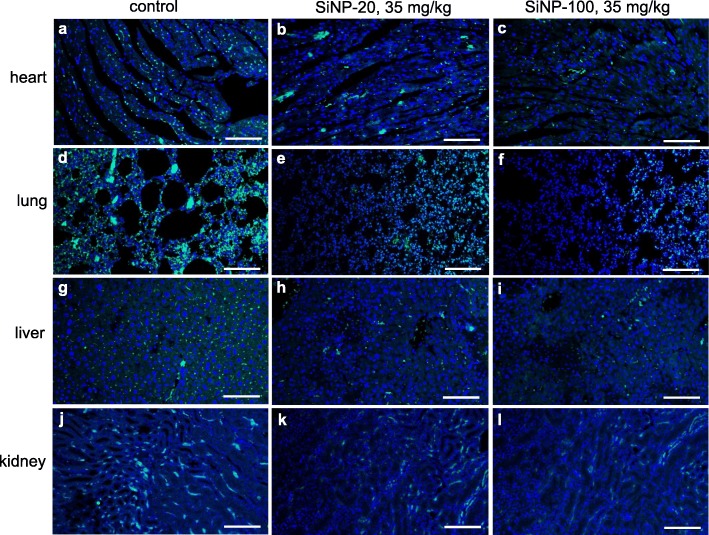
Confocal images showing the toxic effects of SiNPs (35 mg/kg, i.v.) on the expression and spatial localization of VE-cadherin (green) in the heart, lung, liver and kidney of mice in vivo. Nuclei were stained blue with DAPI. Compared to the control tissues which showed a homogenous and dotted pattern of VE-cadherin distribution, mice exposed to SiNP-20 or SiNP-100 for 72 h showed decreased expression, and disrupted distribution, of VE-cadherin, in these organ tissues. Scale bar = 100 μm

We also examined the in vivo potential toxic effects of SiNPs on the expression and the spatial distribution of VE-cadherin in multiple organs of mice using confocal microscopy. Results show that VE-cadherin was abundantly expressed in the heart, lung, liver and kidney of control mice in a homogenous fashion (Fig. 11, left column; Figure S5, first column). SiNP-20 at 7 mg/kg and 21 mg/kg slightly decreased the expression and changed the spatial distribution of VE-cadherin in the above four organs (Figure S5, second and third columns). SiNP-20 at 35 mg/kg significantly decreased the expression of VE-cadherin (Fig. 11, middle column). SiNP-100 at 7, 21 and 35 mg/kg decreased the expression and changed the spatial distribution of VE-cadherin in a manner similar to SiNP-20 (Figure S5, fourth and fifth columns; Fig. 11, right column).

## Discussion

The possible discharge of SiNPs into the environment and increased opportunities for human exposure to this nanomaterial has received considerable attention. Extensive studies on the toxicities of SiNPs are required to deal with this situation. However, existing studies have usually focused on limited aspects of the problem and have not provided sufficient information to adequately know the toxicities of SiNPs at cellular or organismal levels. We used serial cellular and integrative approaches to study endothelial events and damage to several organs after exposure to SiNPs. SiNPs-induced injuries were mainly manifested with the destruction of endothelial barrier and intravascular coagulation. These results increase our understanding of SiNPs toxicity.

Key considerations for characterizing SiNPs toxicity include exposure dosage, dosage extrapolation between animals and humans, the effects of different exposure routes, the cellular effects, and “effective exposure concentration” in vitro. Dosage is obviously a key measure in the toxicity evaluation of nanomaterials. We analyzed and discussed these issues below.

1. The SiNPs concentration in vitro and dosage in vivo. We established a reasonable SiNPs concentration range in the in vitro cell study (0–200 μg/mL), which was smaller than other in vitro SiNPs concentration ranges (0–2000 μg/mL) [10]. The in vivo doses of SiNPs were 0–35 mg/kg (b.w) (i.v.). These were established based on previous studies [21–23]. The highest dose (35 mg/kg) was much lower than the estimated LD50 value of SiNPs (i.v.) in mice (262.45 ± 33.78 mg/kg) [23]. We observed that SiNPs at 7 mg/kg (i.v.) induce little organ injury (histology, shown in Figures S3, S4 and S5 in the Supplementary Material), suggesting that 7 mg/kg SiNPs (i.v.) was a relatively low dose for mice.
2. Dosage extrapolation from mice to humans. The dose range (7–35 mg/kg) in mice is equivalent to a dose range of 0.78–3.88 mg/kg in adult humans based on dose conversion methods between species (in <Methodology of Pharmacological Experiments>; http://www.fda.gov/downloads/Drugs/).
3. Evaluation of nanosilica intake in humans and comparison with animal study. Dekkers et al. [24] reviewed the presence and risks of nanosilica in food products (such as instant noodles, meat, seafood, vegetable rubs, pancakes, asparagus soup, coffee with creamer, and vitamin C pills, etc.). It was estimated that a daily oral nanosilica intake for a 70 kg adult via food could reach 124 mg/day which corresponds to 1.8 mg/kg (b.w.)/day. This value was within the converted adult human dose range (0.78–3.88 mg/kg) from mice dosage (7–35 mg/kg) in the present study.
4. Dose conversion between different exposure routes. In vivo SiNPs exposure routes include inhalation, intravenous administration (usually as a component of drug delivery), ingestion and skin contact. Li et al. [25] calculated a sub-chronic rat inhalation dosage and extrapolated this to humans with reference to the WHO clean air standards. The results indicated that the occupational exposure levels of SiNPs ranged from 1.0 mg/m^3^ to 27.6 mg/m^3^ (air). Sutunkova et al. [26] performed an in vivo long-term toxicology study with realistic SiNPs exposure levels of 2.6 ± 0.6 mg/m^3^ and 10.6 ± 2.1 mg/m^3^ (air). However, the National Institute for Occupational Safety and Health (NIOSH) has not established occupation exposure limits of amorphous SiNPs, and extrapolation of an inhalation dose into an equivalent intravenous dose is an unsolved mathematical problem.
5. The “effective exposure concentration” in vitro. Cell responses depend on the concentration of nanoparticles at the site of action (for example, nanoparticle numbers sedimented onto the cell surface), herein “effective exposure concentration”, rather than the administrated concentration. Therefore, it is best to estimate the effective exposure concentration for an actual experimental setting. The ISDD model [27] and the DG (Distorted Grid) model [28] have been suggested to evaluate the effective exposure concentration. We performed sedimentation analysis for both SiNPs based on the ISDD model. Over time, the larger SiNPs (SiNP-100) sedimented more onto the cell surface than smaller SiNPs (SiNP-20) (Figure S2). This result helps explain why SiNP-100 was more toxic than SiNP-20 as shown in the cell and animal studies.

The key pathology of SiNPs-induced cell and organ injuries is directed at the endothelium. We thus focused on the endothelium and endothelial junctions in the mechanistic studies. SiNPs decreased the viability and damaged the plasma membrane of HUVECs in the CCK8 and LDH assays, and decreased the migration and tube formation abilities of HUVECs in the transwell and matrigel assays in vitro. Damaged endothelial migration and tube formation abilities are harmful to the establishment and maintenance of the endothelial barrier. Red blood cell adhesion to coronary intima and aortic intimal neoplasm formation in vivo also suggests endothelial injury. In a word, SiNPs can severely damage the endothelial cells and endothelial barrier and this endothelial injury is likely the leading pathology of SiNPs toxicity.

We explored the signaling pathway(s), especially the upstream signaling events by which SiNPs damage the endothelium. Calcium mobilization is a common upstream signal in many cellular activities, including the induction of cytotoxicity. However, the potential effects of SiNPs on calcium homeostasis and dynamics in the endothelium are seldom investigated except in other cell types [29]. Endothelial calcium mobilization (elevation of [Ca^2+^]_i_) is determined by Ca^2+^ release from intracellular stores and Ca^2+^ influx through Ca^2+^ entry channels in the plasma membrane. Endothelial cells express at least two different types of Ca^2+^ entry channels: the CRAC channel, which is highly selective to Ca^2+^, and the non-selective Ca^2+^ permeable cation channels (NSC) [30]. Using a fluorescent Ca^2+^ imaging approach, we found that both SiNP-20 and SiNP-100 induced calcium mobilization in HUVECs which was caused partially by Ca^2+^ influx through the CRAC channel. Blocking the CRAC channel with YM58483 significantly reduced, but it did not totally abolish, the calcium mobilization. SiNPs might activate Ca^2+^ releasing channels (such as IP3 receptor) and thus lead to Ca^2+^ release from intracellular stores, depletion of Ca^2+^ stores and activation of Ca^2+^ entry channels and thereafter Ca^2+^ influx. These observations suggested the potential mechanisms by which SiNPs induce endothelial calcium mobilization as follows. First, SiNPs activated the calcium mobilizing machinery of HUVECs at the extracellular side but not at the intracellular side, because we observed that calcium mobilization (cells glowing under the fluorescence microscope) occurred soon after adding SiNPs to the culture dish (within 90 s). This brief period is insufficient to complete the process of SiNP endocytosis, which usually takes at least 20 min. Second, SiNPs might directly activate the CRAC channels and thus induce Ca^2+^ influx which then leads to Ca^2+^ release from intracellular stores via the Ca^2+^ induced Ca^2+^ release (CICR) mechanism. SiNPs-induced Ca^2+^ influx was further confirmed by the NMT approach. Third, SiNPs might also activate Ca^2+^ releasing channels by an unknown mechanism and thus activate CRAC channels via the store depletion mechanism. Overall, the results suggest that calcium mobilization is an upstream signal responsible for the endothelial toxicity of SiNPs.

Crosstalk between Ca^2+^ and ROS has been well established [31, 32]. As SiNPs induced significant calcium mobilization in HUVECs, we expected that this event would also induce ROS generation. We found that both SiNP-20 and SiNP-100 induced significant ROS generation in HUVECs in a concentration-dependent (fluorescence microscopy) and time-dependent (flow cytometry) manner. A flow cytometry experiment showed that the most obvious ROS elevation occurred 2–4 h after exposure to SiNPs. There is a discussion about the specific role of ROS generation in the toxicity of SiNPs. For example, Guo et al. [33] reported that ROS is involved in SiNPs-induced endothelial damage; while Gehrke et al. [34] showed that SiNPs induced cytotoxicity in a human colon carcinoma cell line (HT29) but without induction of ROS. This suggests independence of ROS in the cytotoxicity. Our present study suggests that ROS are signaling molecules mediating the endothelial toxicity of SiNPs, because scavenging ROS by NAC improved the destruction of VE-cadherin (Fig. 6d). Thus, our study supports that the upstream Ca^2+^-ROS signaling is responsible for SiNPs-induced endothelium/organ injuries while VE-cadherin is a downstream signaling molecule of ROS.

VE-cadherin is an endothelial adherens junction protein that plays a major role in the formation of the endothelial barrier [35]. Our results showed that SiNPs suppressed the expression of VE-cadherin and damaged the integrity and continuity of VE-cadherin at the endothelial junctions suggesting destruction of the endothelial barrier. SiNPs also increased the phosphorylation of VE-cadherin at the site of the tyrosine 731 residue (pY731-VEC) in HUVECs, indicating why SiNPs could affect VE-cadherin. We also observed that SiNPs can suppress the expression of VE-cadherin and disrupt the spatial distribution of VE-cadherin in multiple organs in vivo (shown in Fig. 11 and Figure S5).

We showed that SiNPs induced F-actin re-assembly in HUVECs. Actin filaments remodeling has been observed in titanium dioxide nanoparticles and gold nanoparticles that induced endothelial barrier destruction [15, 17]. Actin microfilaments can interact with endothelial adhesion structures and cooperatively regulate the cell shape. F-actin is a major actin protein in the endothelium and it is remodeled in response to nanomaterial exposure, oxidative stress and tumor necrosis factor [15, 36, 37]. Actin is an important component of “stress fibers” [38] and is responsible for endothelial contraction, which may lead to widening of inter-endothelial junctional gap and failure of the endothelial barrier [39]. F-actin remodeling may also suppress endothelial migration and tube formation and further disrupt the endothelial barrier.

HUVECs exposed to SiNPs had a larger cell size (Figs. 6 and 7). This is consistent with the description in the review by Abu Taha and Schnittler [40]. They showed that subconfluent HUVECs cultures exhibited interrupted VE-cadherin and larger cell size. These results suggest that either endothelial cells exposed to SiNPs (our study) or cultured endothelial cells at a subconfluent stage (Abu Taha and Schnittler [40]) are likely to be altered phenotypes with remodeled cell morphology and disruption of VE-cadherin in response to unfavorable conditions.

In vivo experiments show that SiNPs induced multiple organ injuries roughly in a dose-, exposure time-, and particle size-dependent manner. These injuries were similar to the in vitro results. SiNP-100 induced more severe injury than SiNP-20 and a higher dose (35 mg/kg) and longer exposure (72 h) induced more serious injury than lower doses and shorter exposures. Similar to the in vitro results, organ injuries in vivo were also characterized by endothelial injury and pathological changes. For example, heart injury exhibited endothelial injury and red blood cell adhesion to the coronary intima, and coronary coagulation, and these may lead to cardiac ischemia. Lung injury involved pulmonary congestion, smaller pulmonary vein coagulation, bronchiolar epithelial edema, bronchiolar lumen oozing and resultant lumen narrowing or even occlusion, which may cause pulmonary embolism, bronchiolar occlusion, and dyspnea. Liver injury exhibited multifocal necrosis and smaller liver vein coagulation, which may induce hepatic failure. Kidney injury mainly involved smaller kidney vein congestion/coagulation and glomerular congestion and swelling, which may result in renal failure. Macrophage infiltration was observed in all of the observed organ tissues which suggests inflammation secondary to the initial injury and may further aggravate the organ injuries. Some features of SiNPs-induced cell or organ pathologies were seen for the first time here. For example, striking endothelial calcium mobilization, phosphorylation of VE-cadherin, coronary coagulation and neoplasm formation in the abdominal aorta. It is notable that all the animals did not die within the 72-h observation period even those exposed to the largest dose (35 mg/kg) and those with severe organ injuries. The reason might be that the administrated largest dose in the present study was still much lower than the estimated LD50 value for SiNPs (i.v.) in mice (262.45 ± 33.78 mg/kg) reported by Yu et al. [23].

Intravascular coagulation was a striking pathological feature in mice exposed to SiNPs. Coagulation was found mainly in smaller blood vessels, including the arteries and veins in all observed organs, but it was not observed in larger blood vessels such as the aorta. We observed neoplasm in the intima of abdominal aorta. The neoplasm contained karyocytes in its components but was not a coagulated clog. The neoplasm might fall off under the rapid aortic bloodstream and form a new embolism. Endothelial injury and blood exposure to collagen beneath the endothelium can activate factor XII (Hageman factor) and trigger blood coagulation via the intrinsic coagulation pathway (contact pathway) [41]. Intravascular coagulation may be triggered by endothelial injury/exfoliation, collagen exposure, and initiation of the contact pathway after exposure to SiNPs [42], and/or by changing the levels and activities of coagulant factors, anticoagulants and platelets as previously reported [43–45]. Although the pro-coagulation effect of SiNPs was not initially found in the present study, we found impressive intravascular coagulation in multiple organs after in vivo exposure to SiNPs. SiNPs-induced coagulation may lead to serious complications, such as cardiac ischemia, pulmonary embolism, and liver and kidney dysfunction if the coagulation occurs in the blood vessels of these organs.

**Fig. 12:**
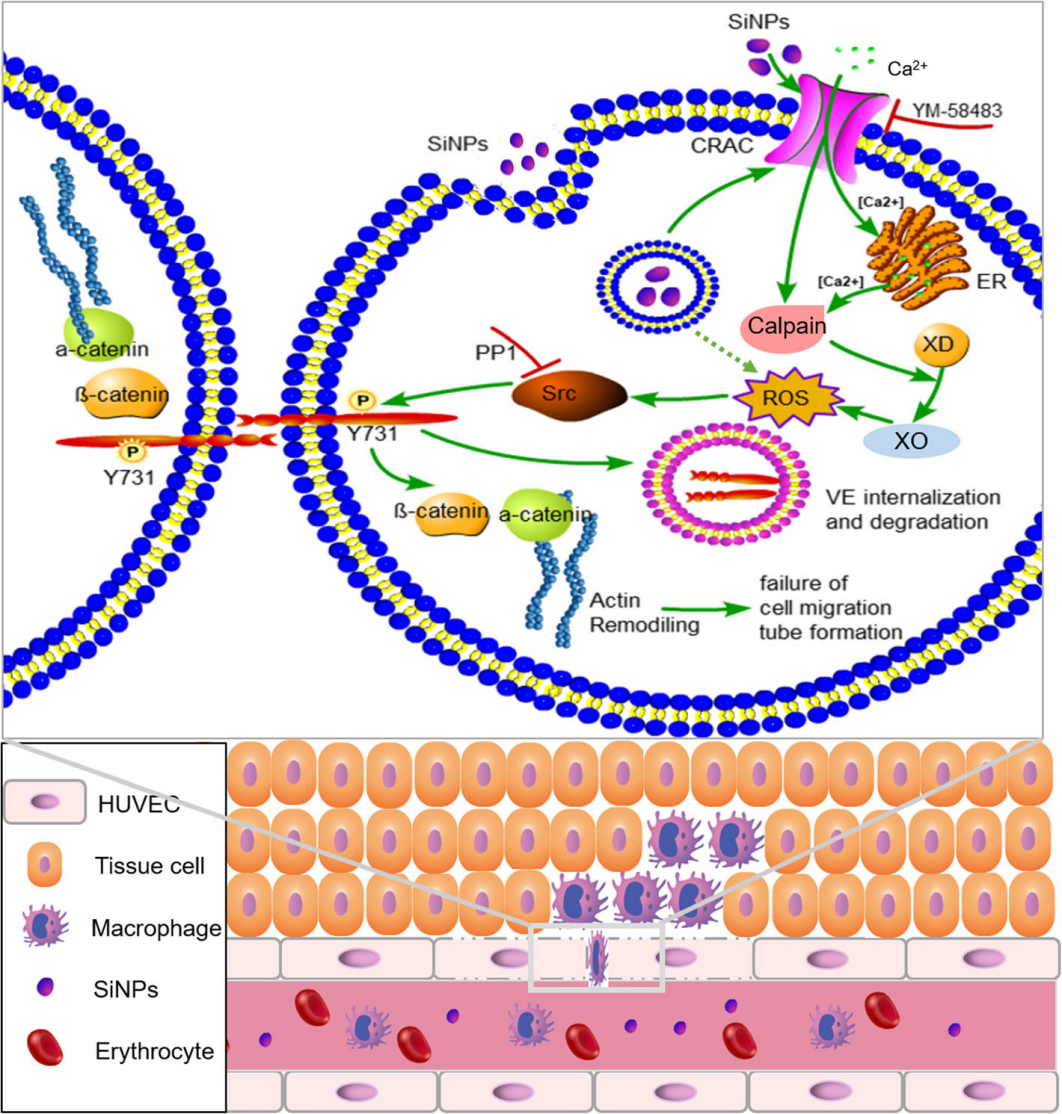
Proposed signaling pathway(s) responsible for SiNPs-induced endothelial barrier injury based on the present study and published literatures. In brief, SiNPs activate CRAC channels at the extracellular side and lead to Ca^2+^ influx (endocytosed SiNPs might also affect the CRAC channel and other proteins from the intracellular side); Ca^2+^ influx induces Ca^2+^ release from the endoplasmic reticulum (ER) via a Ca^2+^-induced Ca^2+^ release (CICR) mechanism; Ca^2+^ influx and Ca^2+^ release lead to [Ca^2+^]_i_ elevation; elevated [Ca^2+^]_i_ activates calpain and convert xanthine dehydrogenase (XD) to xanthine oxidase (XO), and XO catalyzes ROS generation; ROS mediates activation of Src family kinase, and activated Src induces VE-cadherin phosphorylation at the Y731 site and leads to VE-cadherin internalization and degradation and F-actin re-assembly, and finally leads to failure of endothelial barrier. Subsequent macrophage infiltration occurs across the inter-endothelial gap due to the injury. In this signaling chain, MY58483 inhibits CRAC channels and PP1 suppresses Src, and thus together ameliorate SiNPs-induced endothelial barrier injury. In addition, the endothelial toxicity of SiNPs may also lead to endothelial death/exfoliation and collagen exposure, thus trigger the contact pathway of coagulation in the blood vessels (not shown)

We suggest that the signaling pathway through which SiNPs damage the endothelial barrier and induce multiple organ injuries is as follows: SiNPs induce Ca^2+^ mobilization in endothelial cells via Ca^2+^ influx through the CRAC channels and Ca^2+^ releasing channels; elevated [Ca^2+^]_i_ triggers ROS generation; ROS activates the Src family tyrosine kinase; the activated Src tyrosine kinase phosphorylates VE-cadherin at the site of tyrosine 731 residue (pY731-VEC); phosphorylation of VE-cadherin leads to endocytosis and degradation of VE-cadherin and re-assembly of F-actin, and thus results in destruction of endothelial barrier, endothelial cell death/exfoliation and triggering of the contact pathway of coagulation. We summarize this proposed signaling pathway in Fig. 12. Results from the applications of pharmacological tools, including YM58483 (CRAC channel blocker), NAC (ROS scavenger), and PP1 (Src tyrosine kinase inhibitor), supported the proposed signaling pathway. Other studies also support this pathway although alternative nanomaterials and experimental designs were used [15, 17, 38, 39].

Study limitations. The first limitation is that we did not observe the toxic effects of SiNPs on other organs, such as gastrointestinal tract, spleen, pancreas, and brain. A second limitation is that we only studied the toxic effects of SiNPs of two sizes (SiNP-20 and SiNP-100). A third limitation is that we did not measure the phosphorylated level of VE-cadherin in vivo.

## Conclusions

Both SiNP-20 and SiNP-100 induce endothelial injury in vitro and multiple organ injuries in mice in vivo roughly in a concentration/dose, particle size, and exposure time dependent manner. Both SiNPs at up to 35 mg/kg (i.v.) is not lethal in mice exposed for 3 days in vivo. The main pathologies are endothelial barrier destruction, intravascular coagulation, aortic intimal neoplasm formation, liver multifocal necrosis, bronchiolic lumen narrowing, and secondary inflammation. Upstream calcium mobilization and ROS generation and downstream VE-cadherin phosphorylation contribute to endothelial barrier disruption. The study suggests that prevention and treatment of endothelial injury and embolism are important clinical approaches for SiNPs exposure.

## Methods

### Characterization of SiNPs

Silica (SiO_2_) nanoparticles (SiNPs) suspensions were purchased from nanoComposix Company (San Diego, CA, USA). The surfaces of SiNPs were coated with silanol by the manufacturer to increase particle hydrophilicity. The tested SiNPs were with two diameters: 20 nm (SiNP-20) and 100 nm (SiNP-100). The size and morphology of SiNPs were assessed by TEM. The hydrodynamic size distribution and zeta potential of SiNPs in double-distilled water or serum containing medium were measured using Zetasizer (Nano ZS90; Malvern, UK). The SiNPs were sterilized and then dispersed with a sonicator for 5 min prior to use. Potential contamination of endotoxin in the SiNPs solution was also examined using the Limulus amebocyte lysate (LAL) gel clot assay kit (Houshiji Company, Xiamen, China), and endotoxin was proved negative in the SiNPs samples.

### Cell culture

Primary human umbilical vein endothelial cells (HUVECs), endothelial cell culture medium (ECM), and endothelial cell growth supplements were all purchased from Science Cell Research Laboratories (San Diego, CA, USA). Cells were grown in endothelial cell culture medium (ECM) containing 5% fetal bovine serum (FBS) and penicillin-streptomycin endothelial cell growth supplements at 37 °C in a humidified atmosphere containing 5% CO_2_. Cultured HUVECs at 80% confluency were used for further studies.

### Transmission electron microscopy (TEM)

Uptake of SiNPs by HUVECs and intracellular localization of SiNPs after exposure was examined using TEM. Cells were exposed to 50 μg/mL of either SiNP-20 or SiNP-100 and were cultured in ECM at 37 °C in 5% CO_2_ for 24 h. Cells were then washed three times with PBS, detached with 2.5% trpsin, then fixed with 2% glutaraldehyde for 2 h. After dehydration with alcohol, cell samples were embedded in epoxy resin. Ultrathin sections were cut using an ultramicrotome and TEM images of HUVECs were taken under a transmission electron microscope (JEM-100CX; JEOL Ltd., Japan).

### Cytotoxicity assay

Cell viability was assessed using cell count kit (CCK-8, Dojindo Molecular Technologies, Inc., Kumamoto, Japan). HUVECs were seeded into each well of 96-well culture plates at a density of 5 × 10^3^ cells/well. After exposure to SiNPs of either size at concentrations of 0–200 μg/mL for 24 h (*n* = 5), the medium was removed from each well and cells were washed with PBS and incubated for an additional 2 h with fresh medium containing 10 μl of CCK-8 reagent. Then the absorbance of medium was measured at 450 nm using a microplate reader (Synergy-HT, BioTek, Winooski, VT, USA). Data were normalized to the absorbance of the untreated control cells. The viability of control cells was set to 100%.

Cell membrane integrity was assessed by quantifying the lactate dehydrogenase (LDH) released from HUVECs to the medium following pretreatment with SiNPs (0–200 μg/mL) for 24 h using a LDH kit (Nanjing Jiancheng Bioengineering Institute, Nanjing, China). Cell density and experimental grouping were similar with cell viability assay. Each experiment was repeated in 5 parallel wells. Control cells were treated with the culture medium. After incubation for 24 h, the optical density values were measured with a microplate reader.

### Fluorescence microscopy to measure intracellular ROS generation

The level of intracellular reactive oxygen species (ROS) was determined in HUVECs exposed to SiNP-20 and SiNP-100 at concentrations of 25, 50 and 100 μg/mL for 2 h (*n* = 3) using a cell-permeable fluorogenic probe 2,7-dichlorodihydrofluorescein diacetate (DCFH-DA). DCFH-DA diffuses into cells and reacts with ROS to form the highly fluorescent product 2,7-dichlorodihydrofluorescein (DCF). Cells were seeded into a 24-well plate at a density of 1.0 × 10^4^ cells/well and pre-treated with DCFH-DA stock solution for 30 min before exposure to SiNPs. After removing the DCFH-DA solution from all wells, cells were exposed to SiNPs in fresh medium for 2 h. Cells not treated with SiNPs were served as negative control, while cells treated with 7.5 mg/mL H_2_O_2_ were positive control. The fluorescence of oxidized DCF was imaged with a fluorescence microscope (Olympus, Tokyo, Japan).

### Flow cytometry to measure intracellular ROS generation

Intracellular accumulation of ROS in HUVECs after exposure to SiNPs was detected using flow cytometry (FC500; Beckman Coulter, Hialeah, FL, USA). Briefly, HUVECs were seeded into 6-well plates at a density of 2 × 10^5^ cells/dish and were treated with SiNP-20 or SiNP-100 at 100 μg/mL or without SiNPs treatment (control). After incubation with SiNP-20 or SiNP-100 for 1 h, 2 h, 4 h, 6 h, 12 h and 24 h, cells were harvested and dyed with fresh ECM containing DCFH-DA for 20 min at 37 °C, and were then washed thrice with PBS. The production of ROS was determined using flow cytometry with an emission wavelength of 525 nm (FL1) for DCF. Data were calculated using CellQuestR software (Becton Dickinson, Mountain View, CA, USA) and expressed as mean channel fluorescence for each sample (1 × 10^4^ cells auto-selected from 2 × 10^6^ cells by flow cytometer).

### Transwell assay

HUVECs migration was evaluated using a transwell system (Corning Costar Corp., Cambridge, MA, USA) which comprised 8 μm polycarbonate filter inserts in 24-well plates. Briefly, cells were trypsin-harvested in ECM with 5% FBS. Next, 600 μL of medium containing 5% FBS without or with SiNP-20 and SiNP-100 at concentrations of 25, 50 and 100 μg/mL was added to the lower chambers, while HUVECs (5 × 10^4^ cells) were plated in the upper chambers. After 12 h incubation, cells migrated to the bottom side of the transwell membrane were fixed with 4% paraformaldehyde at 37 °C for 15 min and stained with 1% crystal violet at 37 °C for 20 min. The non-migrating cells in the upper chamber were removed with blunt-end swabs. The membranes were washed three times with PBS and photographed under a fluorescence microscope (Olympus; Tokyo, Japan). The amount of cell migration was counted under 5 fields. Each treatment was repeated for 3 independent chambers.

### Capillary-like tube formation assay

HUVECs tended to form capillary-like structures (tubes) on matrigel. Tube formation was observed and evaluated with confocal microscopy. HUVECs were treated with SiNP-20 and SiNP-100 at concentrations of 25, 50 and 100 μg/mL or without SiNPs treatment (control). Each treatment was repeated for 3 independent dishes. At least 30 min before the experiment, a special dish for confocal study was coated with matrigel (BD Biosciences, Bedford, MA, USA). Next, trypsin-harvested HUVECs were seeded onto the plated matrigel (2 × 10^5^ cells/well) in cell culture medium and incubated at 37 °C for 6 h. After dying with Cell Tracker Green (Invitrogen, Carlsbad, CA, USA) for 30 min, images of capillary-like structures were captured under a laser scanning confocal microscope (Olympus, Tokyo, Japan). Tubular structures were quantified by manually counting the numbers of connected cells in randomly selected fields at 400× magnification.

### Intracellular free calcium imaging

HUVECs were seeded into glass-bottom culture dishes at a density of 2 × 10^5^ cells/dish and were incubated overnight for adhesion. The next day, after incubation with fresh ECM containing Fura-4 AM for 30 min at 37 °C, cells were washed thrice with PBS. All calcium fluorescent images were conducted under an Olympus IX70 microscope with a CCD camera controlled by MetaFluor software (Universal Imaging Corporation, Downingtown, PA, USA). Images were captured every 3 s. After recording the baseline [Ca^2+^]_i_ for a period of time, both SiNPs at a concentration of 100 μg/mL were added acutely into the dish in the presence or absence of YM-58483 (CRAC inhibitor). The magnitudes of Ca^2+^ transients induced by SiNPs were represented by the Ca^2+^ indicator fluorescence intensity and expressed as the ratio of the fluorescence (F/F0) relative to the resting fluorescence (F0).

### Non-invasive micro-test to measure transmembrane Ca^2+^ flux

Non-invasive micro-test (NMT) technology was applied to measure net Ca^2+^ flux near the plasma membrane of HUVECs using the NMT100 Series (Younger USA LLC, Amherst, MA, USA) equipped with Ca^2+^-sensitive microelectrodes. This technique allows to non-invasively obtain the dynamic information of ionic or molecular activities on material surfaces [46]. Briefly, HUVECs were treated with the same way as that for intracellular calcium imaging. The Ca^2+^-sensitive microelectrode was filled with liquid calcium ion-exchanger and calibrated to keep the slop of [Ca^2+^] change within the standard range. HUVECs were rinsed with external solution composed of (in mmol/L) 120 NaCl, 3 KCl, 2.5 CaCl_2_, 1 MgCl_2_, 1.25 NaH_2_PO_4_, 25 NaHCO_3_ and 10 glucose. The microelectrode was positioned to 5 μm near the cell surface and then was moved slightly back and forth controlled by the software. Ca^2+^ flux data were acquired at a sampling rate of 1 point per 6 s. After 3 min baseline recording, SiNPs of either size at a concentration of 100 μg/mL was acutely added to the dish, and the effects of SiNPs on Ca^2+^ flux were recorded real-timely and were analyzed using the software. Blank control (without cell) and positive control (high K^+^, 60 mmol/L) experiments of NMT were also performed in HUVECs.

### Dosimetry modeling in vitro

The sedimentation levels of SiNPs onto the cell surface in vitro, which indicate the “effective exposure concentration”, was computationally estimated by the in vitro sedimentation, diffusion and dosimetry (ISDD) model, following treatment with either SiNP-20 or SiNP-100 [27, 47]. The model is available as Matlab code from its developers [27], and can be downloaded from http://nanodose.pnnl.gov/ModelDownload.aspx. Further details and modeling parameters can be found in the legend of Figure S2.

### Animals and in vivo experiments

Female Balb/c mice (8 weeks old, 20–22 g body weight) were purchased from the Experimental Animal Center of Shanxi Medical University (Taiyuan, China). Animals were fed with regular chow and water ad libitum and maintained in 12:12 h light/dark luminosity cycles. Animal experiments were performed to examine the potential hazardous effects of SiNPs on organs in vivo. SiNPs were diluted in 200 μl saline and intravenously (i.v.) injected at 7, 21, and 35 mg/kg (body weight) [21–23]. Same volume saline injection was used as controls. Animals were randomly divided to three groups according to treatment: SiNP-20, SiNP-100, and control (*n* = 6 in each group). Mice were painlessly sacrificed 72 h after injection, organ tissues, including heart, abdominal aorta, lung, liver and kidney, were harvested and morphology changes were examined.

### Histochemistry and immunohistochemistry

The tissue samples of heart, aorta, lung, liver and kidney were fixed in 10% formalin, embedded in paraffin, sectioned (5 μm) and attached to slides, deparaffinized, and stained with hematoxylin and eosin (H&E) to generally check the tissue structural changes after exposure to SiNPs. To perform immunohistochemical staining of F4/80 (a macrophage marker) in organ tissues after exposure to SiNPs in vivo, tissue sections were reacted with a 3% hydrogen peroxide/methanol solution to inactivate endogenous peroxidase, washed with PBS, and treated with antigen-unmasking reagent. Tissue sections were then blocked with 10% normal goat serum for 10 min and then incubated with the primary antibody against F4/80 (dilution 1:50) (Abcam, Cambridge, UK) for overnight at 4 °C. The sections were washed with PBS and incubated with avidin-biotin conjugated secondary antibody for 30 min at room temperature, then washed with PBS and reacted with DAB substrate followed by wash with water. Immunoreactive signals in the sections were shot under a microscope.

### Immunofluorescent staining

Immunofluorescent staining was used to examine the effects of SiNPs on the expression and localization of junctional protein VE-cadherin and cytoskeleton protein F-actin in HUVECs in vitro and VE-cadherin in tissues in vivo.

To perform cell staining, HUVECs were seeded into glass-bottom culture dishes at a density of 2 × 10^5^ cells/dish and were incubated overnight for adhesion. Cells were then incubated with both SiNPs at 100 μg/mL in 1 mL culture medium for 2 h. Control cells were treated with same volume of PBS instead of SiNPs. Cells were then fixed with 4% paraformaldehyde for 10 min at 37 °C. After rinsing with fresh PBS, cells were incubated with goat serum for 1 h at room temperature. Cells were then labeled with VE-cadherin XPTM rabbit antibody (dilution 1:200) (Cell Signaling Technology, Danvers, MA, USA) in goat serum at 4 °C for overnight. Thereafter, cells were washed three times with PBS and incubated for 1 h at room temperature with Alexa Fluor®488-labeled goat anti-rabbit antibody (dilution 1:400) (Invitrogen, Carlsbad, CA, USA). F-actin was stained with TRITC-phalloidin (Shanghai Solarbio Bioscience & Technology Co., Ltd., Shanghai, China) additionally. Cells were washed with PBS and mounted with an aqueous mounting medium containing DAPI (Zhongshan Golden bridge biotechnology Co., Beijing, China). Cell fluorescent images were taken using the FluoView FV1000 confocal microscope (Olympus, Tokyo, Japan). The positive fluorescent signals were analyzed using FluoView software (Olympus, Tokyo, Japan).

To perform tissue immunofluorescent staining, tissues were routinely fixed with 10% formalin, dehydrated and embedded in paraffin. Five μm sections were cut with a microtome and mounted on slides, then were deparaffinized and rehydrated and treated with antigen-unmasking reagent. Sections were blocked with 10% normal goat serum for 10 min and then incubated with VE-cadherin XPTM rabbit antibody (dilution 1:200) (Cell Signaling Technology, Danvers, MA, USA) for overnight at 4 °C. The following procedures were the same as that described above in cell immunofluorescent staining. Positive immunofluorescent signals in the tissue sections were captured under a fluorescence microscope.

### Western blotting

Western blotting was used to detect the expression levels of VE-cadherin and phosphorylated VE-cadherin (pY731-VEC) in HUVECs after exposure to SiNPs for 6 h. Proteins were quantified with bicinchoninic acid assay (Pierce, Rockford, IL, USA). Equal amounts of proteins were loaded onto a sodium dodecyl sulfate (SDS)-polyacrylamide gel and electrophoretically transferred to a polyvinylidene fluoride membrane (Millipore, Billerica, MA, USA), then 5% nonfat milk in tris-buffered saline (TBS) was applied to block the membrane for 1 h. The membrane was incubated with the primary anti-VE-cadherin XPTM antibody (dilution 1:1000) or anti-pY731-VEC antibody (dilution 1:500) for overnight at 4 °C. The membrane was then washed thrice with TBS and Tween 20 (TBST) and incubated with a horseradish peroxidase-conjugated anti-rabbit immunoglobulin G secondary antibody (Zhongshan Golden bridge biotechnology Co., Beijing, China) for 1 h at room temperature. After washing thrice with TBST, the positive target signals were detected using enhanced chemiluminescence substrate (Boster Biological Technology, Wuhan, China). Analysis of the protein bands was performed using ImageLab™ Software (Bio-Rad, Hercules, CA, USA).

### Statistical analysis

Data were presented as mean ± standard deviation (SD). Statistical differences between variant treatments were analyzed with the independent sample-test using IBM SPSS Statistic 19 software. The normal distribution of data was tested prior to performing the *t*-test. Multiple group comparison was performed using analysis of variance. Differences were considered significant at *p* < 0.05.

## Supplementary information

**Additional file 1: Figure S1.** Blank control (without cell) (**a**) and positive control (high K^+^) (**b**) of the NMT experiments in HUVECs in vitro. Adding SiNPs to the dish solution did not yield obvious biological Ca^2+^ flux signal but a small mechanical disturb signal was seen in the blank control experiment (**a**). High K^+^ (60 mmol/L) induced transient Ca^2+^ influx in a HUVEC by depolarization (**b**). **Figure S2.** Transport rates for silica nanoparticles in vitro. The deposition fractions of SiNP-20 and SiNP-100 were calculated using the ISDD model after delivering these nanoparticles to cell culture medium over a duration of 24 h. Note that the deposition fraction of SiNP-100 was higher than SiNP-20 after exposure for ≥15 h. Parameters included the hydrodynamic diameter measured by DLS, medium column height (3.1 mm), temperature (310 K), medium density (1.00 g/cm^3^), and medium dynamic viscosity (0.00074 Pa·s). **Figure S3.** H&E stains of multiple organ tissues showing the toxic effects of SiNP-20 and SiNP-100 at lower doses (7 and 21 mg/kg, i.v.) and exposure for 72 h in mice in vivo. Scal bar = 100 μm for all subpanels. **Figure S4.** Immunohistochemical stains of F4/80 (macrophage marker, brown) in several organ tissues in vivo which reflect macrophage infiltration in response to lower doses of SiNPs (7 and 21 mg/kg, i.v.). Both SiNPs at 7 mg/kg almost did not induce macrophage infiltration, while at 21 mg/kg induced substantial macrophage infiltration. Scal bar = 50 μm. **Figure S5.** Confocal images of organ tissues showing the effects of lower doses of SiNP-20 and SiNP-100 (7 and 21 mg/kg, i.v.) on the expression and spatial distribution of VE-cadherin (green) in multiple organ tissues in vivo. Scale bar = 100 μm.

## Data Availability

All data generated or analyzed during this study are included in this published article.

## Abbreviations

CRAC: Ca^2+^ release activated Ca^2+^ (channel)
DCFH-DA: 2′,7′-dichlorodihydrofluorescein diacetate
HUVECs: Human umbilical vein endothelial cells
LDH: Lactate dehydrogenase
NAC: N-acetyl-L-cysteine
NMT: Non-invasive micro-test
ROS: Reactive oxygen species
SiNP-100: Silica nanomaterials with a diameter of 100 nm
SiNP-20: Silica nanomaterials with a diameter of 20 nm
SiNPs: Silica nanomaterials
TEM: Transmission electron microscopy
VE: Vascular endothelium
