# Supplementary material for: Silica nanomaterials induce organ injuries by Ca2+-ROS-initiated disruption of the endothelial barrier and triggering intravascular coagulation
Source: Part Fibre Toxicol. 2020 Mar 23;17:12. doi: 10.1186/s12989-020-00340-8 (PMC7087393; doi:10.1186/s12989-020-00340-8)
Supplement: Supplementary file 1 — Additional file 1: Figure S1. Blank control (without cell) (a) and positive control (high K+) (b) of the NMT experiments in HUVECs in vitro. Adding SiNPs to the dish solution did not yield obvious biological Ca2+ flux signal but a small mechanical disturb signal was seen in the blank control experiment (a). High K+ (60 mmol/L) induced transient Ca2+ influx in a HUVEC by depolarization (b). Figure S2. Transport rates for silica nanoparticles in vitro. The deposition fractions of SiNP-20 and SiNP-100 were calculated using the ISDD model after delivering these nanoparticles to cell culture medium over a duration of 24 h. Note that the deposition fraction of SiNP-100 was higher than SiNP-20 after exposure for ≥15 h. Parameters included the hydrodynamic diameter measured by DLS, medium column height (3.1 mm), temperature (310 K), medium density (1.00 g/cm3), and medium dynamic viscosity (0.00074 Pa·s). Figure S3. H&E stains of multiple organ tissues showing the toxic effects of SiNP-20 and SiNP-100 at lower doses (7 and 21 mg/kg, i.v.) and exposure for 72 h in mice in vivo. Scal bar = 100 μm for all subpanels. Figure S4. Immunohistochemical stains of F4/80 (macrophage marker, brown) in several organ tissues in vivo which reflect macrophage infiltration in response to lower doses of SiNPs (7 and 21 mg/kg, i.v.). Both SiNPs at 7 mg/kg almost did not induce macrophage infiltration, while at 21 mg/kg induced substantial macrophage infiltration. Scal bar = 50 μm. Figure S5. Confocal images of organ tissues showing the effects of lower doses of SiNP-20 and SiNP-100 (7 and 21 mg/kg, i.v.) on the expression and spatial distribution of VE-cadherin (green) in multiple organ tissues in vivo. Scale bar = 100 μm. [file 12989_2020_340_MOESM1_ESM.docx]

**Supplementary Material**

**Silica nanomaterials induce organ injuries by Ca^2+^-ROS-initiated disruption of the endothelial barrier and triggering intravascular coagulation**

De-Ping Wang^1,*^, Zhao-Jun Wang^1,*^, Rong Zhao^1^, Cai-Xia Lin^1^, Qian-Yu Sun^1^, Cai-Ping Yan^2^, Xin Zhou^3,#^, Ji-Min Cao^1,#^

1. Key Laboratory of Cellular Physiology at Shanxi Medical University, Ministry of Education, and the Department of Physiology, Shanxi Medical University, Taiyuan, China

2. Center of Translational Medicine, Shanxi Medical University, Taiyuan, China

3. Department of Medical Imaging, Shanxi Medical University, Taiyuan, China

^#^ These authors contributed to this work.

^*^ Corresponding Authors. Email: zhouxin_1106@163.com (XZ); [caojimin@126.com](mailto:caojimin@126.com) (JC)

**Supplementary figures and legends**

**
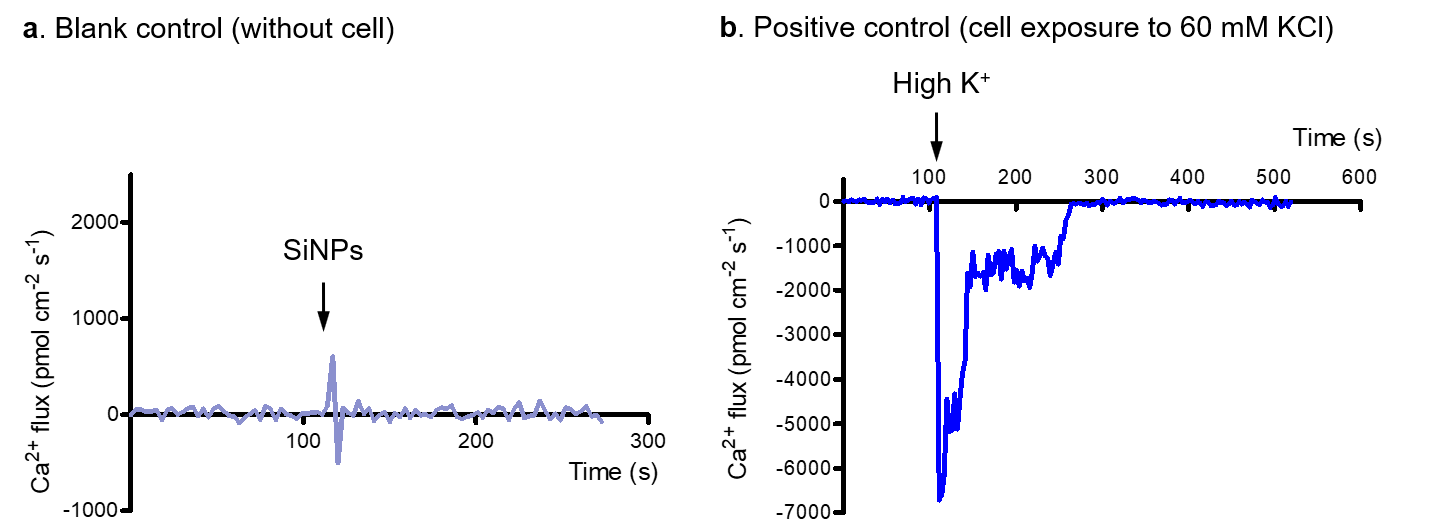
**

**Fig. S1** Blank control (without cell) (**a**) and positive control (high K^+^) (**b**) of the NMT experiments in HUVECs *in vitro*. Adding SiNPs to the dish solution did not yield obvious biological Ca^2+^ flux signal but a small mechanical disturb signal was seen in the blank control experiment (**a**). High K^+^ (60 mmol/L) induced transient Ca^2+^ influx in a HUVEC by depolarization (**b**).

**
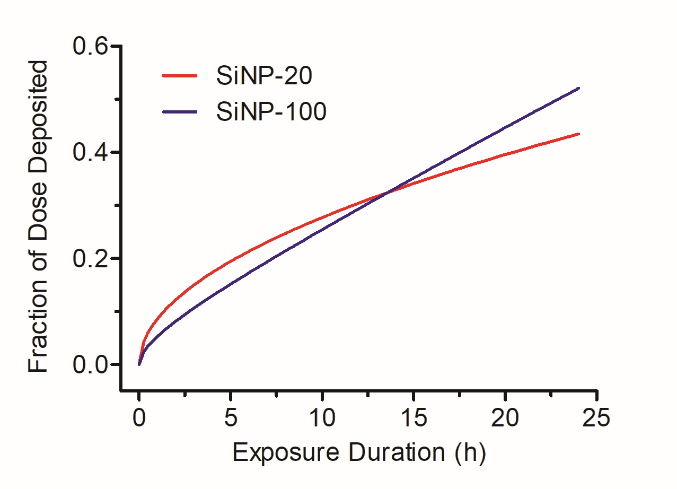
**

**Fig. S2** Transport rates for silica nanoparticles *in vitro*. The deposition fractions of SiNP-20 and SiNP-100 were calculated using the ISDD model after delivering these nanoparticles to cell culture medium over a duration of 24 h. Note that the deposition fraction of SiNP-100 was higher than SiNP-20 after exposure for ≥15 h. Parameters included the hydrodynamic diameter measured by DLS, medium column height (3.1 mm), temperature (310 K), medium density (1.00 g/cm^3^), and medium dynamic viscosity (0.00074 Pa·s).

**
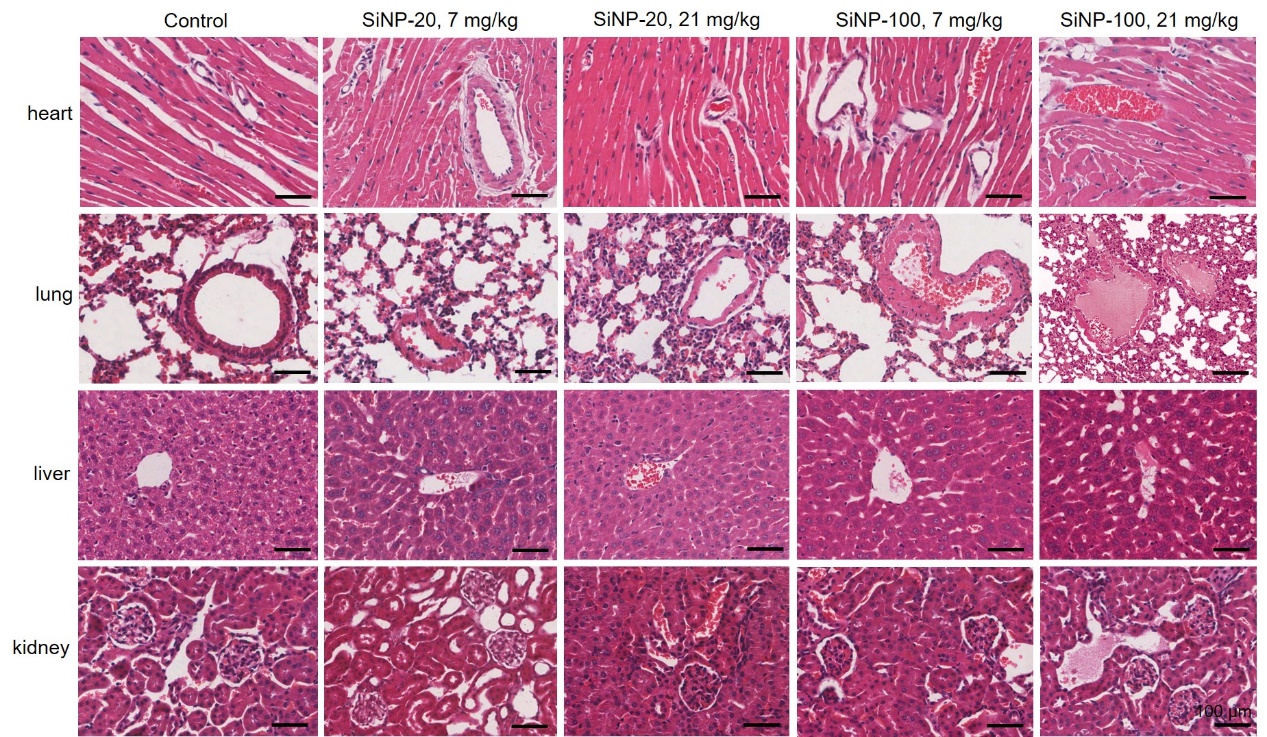
**

**Fig. S3** H&E stains of multiple organ tissues showing the toxic effects of SiNP-20 and SiNP-100 at lower doses (7 and 21 mg/kg, i.v.) and exposure for 72 h in mice *in vivo*. Scal bar = 100 μm for all subpanels.

**
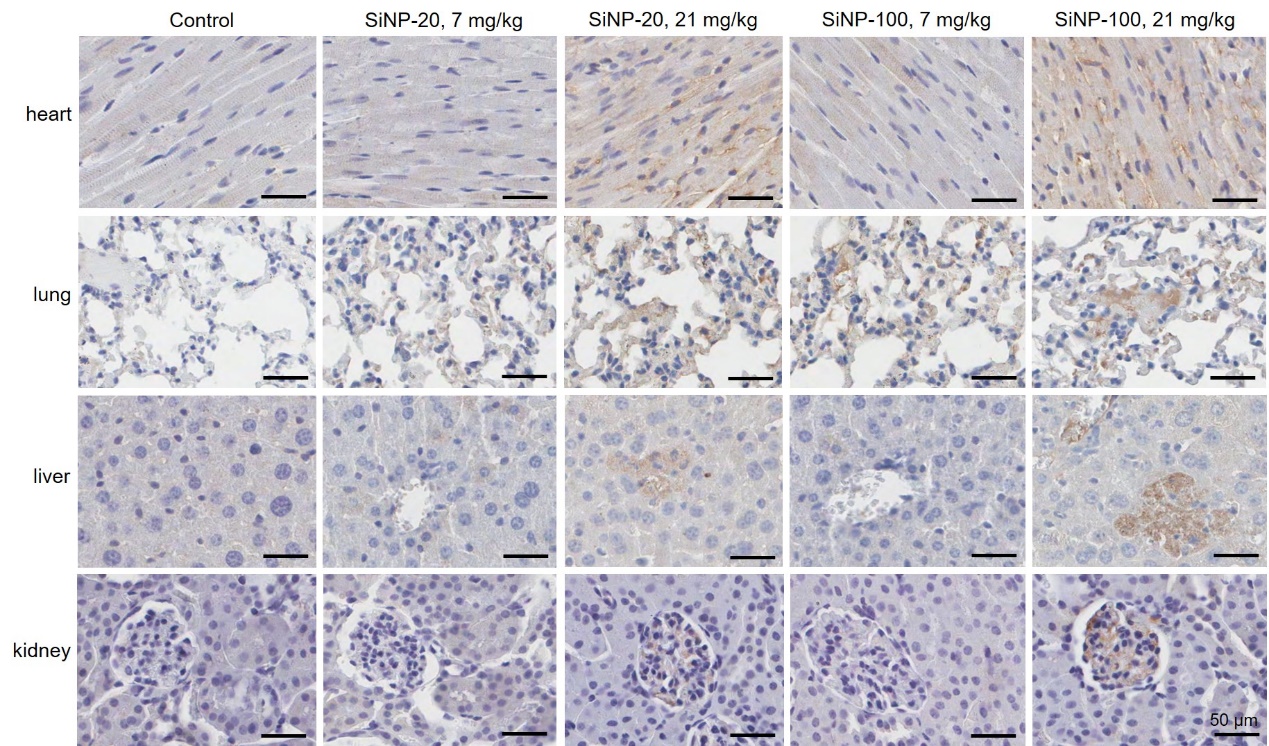
**

**Fig. S4** Immunohistochemical stains of F4/80 (macrophage marker, brown) in several organ tissues *in vivo* which reflect macrophage infiltration in response to lower doses of SiNPs (7 and 21 mg/kg, i.v.). Both SiNPs at 7 mg/kg almost did not induce macrophage infiltration, while at 21 mg/kg induced substantial macrophage infiltration. Scal bar = 50 μm.


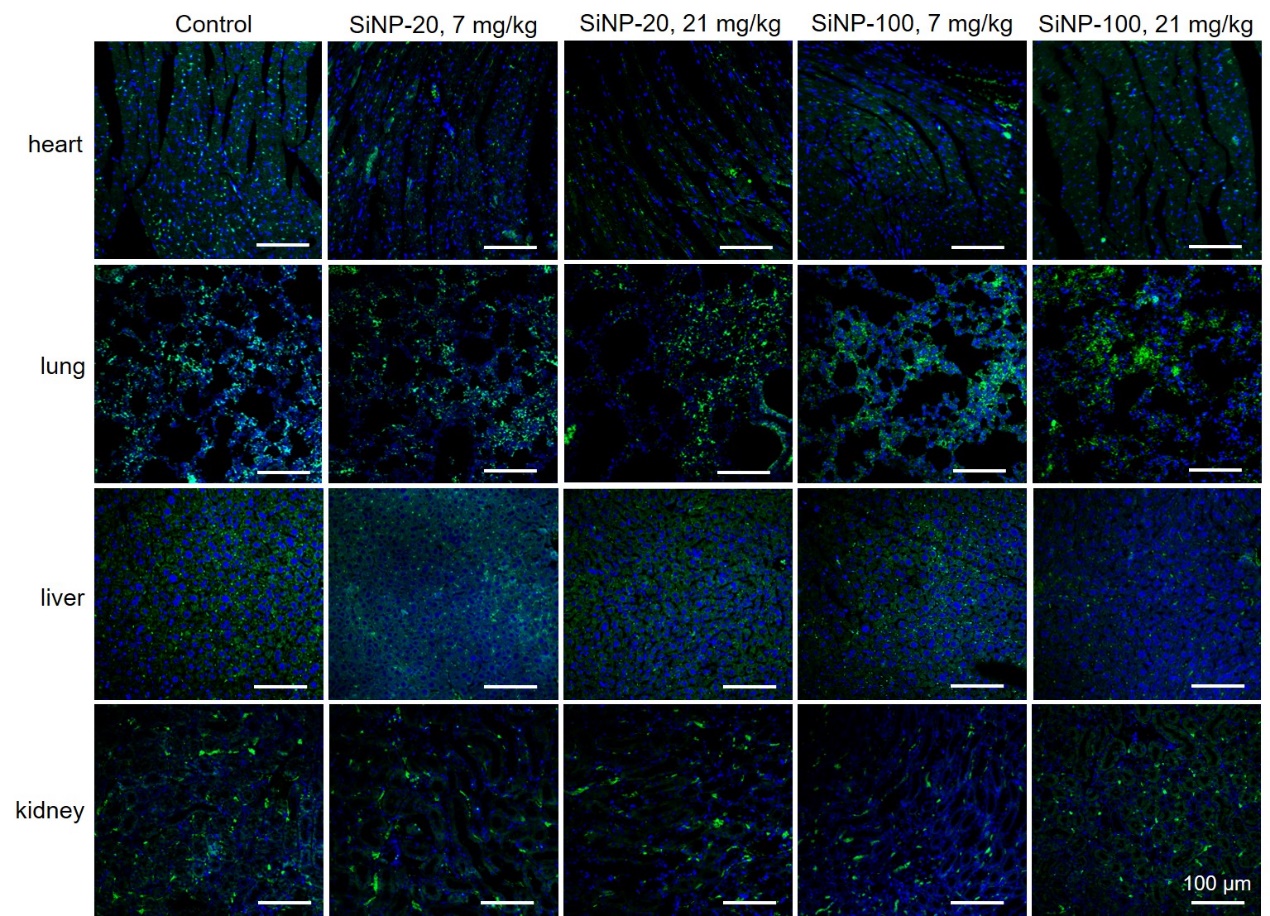


**Fig. S5** Confocal images of organ tissues showing the effects of lower doses of SiNP-20 and SiNP-100 (7 and 21 mg/kg, i.v.) on the expression and spatial distribution of VE-cadherin (green) in multiple organ tissues *in vivo*. Scale bar = 100 μm.
